# Unveiling the spatial distribution and molecular mechanisms of terpenoid biosynthesis in *Salvia miltiorrhiza* and *S. grandifolia* using multi-omics and DESI–MSI

**DOI:** 10.1093/hr/uhad109

**Published:** 2023-05-31

**Authors:** Jie Xia, Ganggui Lou, Lan Zhang, Yanbo Huang, Jian Yang, Juan Guo, Zhechen Qi, Zhenhao Li, Guoliang Zhang, Shengchun Xu, Xijiao Song, Xiaodan Zhang, Yukun Wei, Zongsuo Liang, Dongfeng Yang

**Affiliations:** College of Life Sciences and Medicine, Key Laboratory of Plant Secondary Metabolism and Regulation of Zhejiang Province, Zhejiang Sci-Tech University, 310000, Hangzhou, China; College of Life Sciences and Medicine, Key Laboratory of Plant Secondary Metabolism and Regulation of Zhejiang Province, Zhejiang Sci-Tech University, 310000, Hangzhou, China; College of Life Sciences and Medicine, Key Laboratory of Plant Secondary Metabolism and Regulation of Zhejiang Province, Zhejiang Sci-Tech University, 310000, Hangzhou, China; Eastern China Conservation Centre for Wild Endangered Plant Resources, Shanghai Chenshan Botanical Garden, 200000, Shanghai, China; State Key Lab Breeding Base Dao-Di Herbs, National Resource Center Chinese Materia Medica, Beijing, China Academy of Chinese Medical Sciences, 100000, Beijing, China; State Key Lab Breeding Base Dao-Di Herbs, National Resource Center Chinese Materia Medica, Beijing, China Academy of Chinese Medical Sciences, 100000, Beijing, China; College of Life Sciences and Medicine, Key Laboratory of Plant Secondary Metabolism and Regulation of Zhejiang Province, Zhejiang Sci-Tech University, 310000, Hangzhou, China; Zhejiang Shouxiangu Botanical Drug Institute Co., Ltd, 310000, Hangzhou, China; Zhejiang Shouxiangu Botanical Drug Institute Co., Ltd, 310000, Hangzhou, China; State Key Laboratory for Managing Biotic and Chemical Threats to the Quality and Safety of Agro-products, Zhejiang Academy of Agricultural Sciences, 310000, Hangzhou, China; State Key Laboratory for Managing Biotic and Chemical Threats to the Quality and Safety of Agro-products, Zhejiang Academy of Agricultural Sciences, 310000, Hangzhou, China; College of Life Sciences and Medicine, Key Laboratory of Plant Secondary Metabolism and Regulation of Zhejiang Province, Zhejiang Sci-Tech University, 310000, Hangzhou, China; Shanghai Botanical Garden, Shanghai, China; College of Life Sciences and Medicine, Key Laboratory of Plant Secondary Metabolism and Regulation of Zhejiang Province, Zhejiang Sci-Tech University, 310000, Hangzhou, China; College of Life Sciences and Medicine, Key Laboratory of Plant Secondary Metabolism and Regulation of Zhejiang Province, Zhejiang Sci-Tech University, 310000, Hangzhou, China

## Abstract

*Salvia miltiorrhiza* and *S. grandifolia* are rich in diterpenoids and have therapeutic effects on cardiovascular diseases. In this study, the spatial distribution of diterpenoids in both species was analyzed by a combination of metabolomics and mass spectrometry imaging techniques. The results indicated that diterpenoids in *S. miltiorrhiza* were mainly abietane-type norditerpenoid quinones with a furan or dihydrofuran D-ring and were mainly distributed in the periderm of the roots, e.g. cryptotanshinone and tanshinone IIA. The compounds in *S. grandifolia* were mainly phenolic abietane-type tricyclic diterpenoids with six- or seven-membered C-rings, and were widely distributed in the periderm, phloem, and xylem of the roots, e.g. 11-hydroxy-sugiol, 11,20-dihydroxy-sugiol, and 11,20-dihydroxy-ferruginol. In addition, the leaves of *S. grandifolia* were rich in tanshinone biosynthesis precursors, such as 11-hydroxy-sugiol, while those of *S. miltiorrhiza* were rich in phenolic acids. Genes in the upstream pathway of tanshinone biosynthesis were highly expressed in the root of *S. grandifolia*, and genes in the downstream pathway were highly expressed in the root of *S. miltiorrhiza*. Here, we describe the specific tissue distributions and mechanisms of diterpenoids in two *Salvia* species, which will facilitate further investigations of the biosynthesis of diterpenoids in plant synthetic biology.

## Introduction

*Salvia miltiorrhiza* Bunge is an important species of the genus *Salvia* (sage), which has extremely great economic and medicinal value [1]. The chemical constituents of *S. miltiorrhiza* are mainly diterpenoid tanshinones and polyphenolic salvianolic acids, such as tanshinone IIA, cryptotanshinone, salvianolic acid B, and rosmarinic acid. Pharmacological studies in recent years have shown that tanshinones and other components of *S. miltiorrhiza* possess a broad range of activities [2–4]. For example, in a variety of human tumor cell lines, tanshinone IIA has exhibited a wide range of antitumor activities, such as tumor growth inhibition, the induction of apoptosis, cell cycle regulation, the modulation of signaling pathway-inducing factors, and the reversal of multidrug resistance [5, 6]. In a mouse model of reactive gliosis and neuroinflammation, cryptotanshinone exhibited anti-inflammatory and neuroprotective effects that inhibited the onset and progression of Alzheimer’s disease [7]. In addition, salvianolic acids have strong antioxidant effects and thereby regulate redox homeostasis, reduce the activity of redox-activated enzymes, and inhibit cellular stress and apoptosis [8, 9], while hemicellulose-basedpolysaccharides are not cytotoxic and also have the potential to become antitumor agents [10].

*Salvia grandifolia* W. W. Sm. is a perennial plant endemic to China. It is mainly distributed in southwestern China and is often used as a substitute for *S. miltiorrhiza* in Sichuan and Yunnan provinces [11]. The lipophilic components of *S. grandifolia* are similar to those of *S. miltiorrhiza* and are mainly diterpenoids, but the skeletal structures of some diterpenoids in *S. grandifolia* are distinct from those of the tanshinones in *S. miltiorrhiza*. For example, grandifolia B is a diterpenoid with a rare seven-membered C-ring [12, 13]. Over the past decade, we have collected >100 species of *Salvia* in East Asia and globally, and have found that only the phloem and xylem of *S. grandifolia* roots show significant yellow and red color, whereas all of the tanshinones we have analyzed are red or yellow in color (Supplementary Data Fig. S1). In an extensive study of *S. miltiorrhiza*, red tanshinones were only found in the periderm. Thus, it remains unclear whether these red substances in the phloem and xylem of *S. grandifolia* roots are tanshinones or their derivatives. Moreover, it is not clear whether *S. miltiorrhiza* and *S. grandifolia* have different diterpenoid skeletons or diterpenoid distributions.

Diterpenoids are widely distributed in *Salvia* species, and the biosynthetic pathways of diterpenoids, such as tanshinones, have attracted an increasing amount of research attention due to their multiple biological activities. Advances in high-throughput sequencing and bioinformatics analysis techniques have provided a large number of candidate gene-expression profiles for elucidating the synthetic pathways of naturally occurring products [14]. Diterpenoids are usually derivatives of the linear 20-carbon diterpenoid (*E*,*E*,*E*)-geranylgeranyl diphosphate (GGPP), and diterpenoid synthases/cyclases mediate the cyclization and/or rearrangement of GGPP to form a superfamily of diterpenoids with various hydrocarbon skeletal structures [15]. First, the labdadienyl/copalyl diphosphate synthase SmCPS1 cyclizes GGPP to a copolymer diphosphate (CPP), which the class I diterpene synthase SmKSL1 then cyclizes and rearranges to afford abietane miltiradiene [16]. By screening candidate cytochrome 450 (CYP) genes co-regulated with diterpenoid synthase genes using next-generation sequencing, it was demonstrated that CYP76AH1 catalyzes the hydroxylation of C11 of miltiradiene to form ferruginol both *in vitro* and *in vivo* [17]. In addition, genome assembly analysis of the *Salvia* cell line bh2-7 determined that CYP71D373 and CYP71D375 directly catalyze the formation of a typical furan D-ring [18]. Recent studies have shown that TIIA synthase (SmTIIAS) is a dehydrogenase that catalyzes the aromatization of the dihydrofuran precursors to form the furan ring in these compounds, and controls the flow of metabolites from dihydrofuran to furan-tanshinone, resulting in the accumulation of dihydrofuran-tanshinone and a decrease in the production of furan-tanshinone. Thus, the metabolic pathway for the conversion of cryptotanshinone to tanshinone IIA has also been resolved [19]. However, although the above-mentioned studies have identified the catalytic enzymes and synthetic pathways associated with furan-type diterpenes and furan-D-ring-containing diterpenes found in members of the Lamiaceae family, many intermediate catalytic modification processes remain unclear.

Mass spectrometry imaging (MSI) is a powerful tool that can directly characterize the chemical characteristics and spatial distribution of different samples without any chemical modification or labeling [20]. It combines qualitative and quantitative molecular information with spatial information, mapping specific molecules to the specific tissue distribution of the original sample, complementing traditional chemical analysis [21]. At the same time, MSI combined with other analytical techniques greatly expands the understanding of sample information and is crucial for elucidating the synthesis, accumulation, and regulation mechanisms of endogenous substances [20]. Recently, Dong *et al*. combined MSI with different reverse genetic approaches to elucidate the gene functions of specific genes in tomato fruit, *Nicotiana benthamiana* leaves, and wheat epicuticular waxes, tissues or cells [22]. Additionally, Li *et al*. used MSI and metabolome analysis to investigate the spatial distribution characteristics of key secondary metabolites in the roots of two species of *Paeonia* plants [23].

In this study we combined multi-omics and spatial metabolomics with high-resolution mass spectrometry, and used this method to analyze the spatial distributions of the main active metabolites in root and leaf tissues of *S. miltiorrhiza* and *S. grandifolia*. The system parameters and sample preparation methods were optimized to accurately locate the main diterpenoid tanshinones, phenolic acids, polysaccharides, and glycosides, and analyze the main endogenous substances in leaves. Through a combination of tanshinone-associated integrated metabolite and transcriptome analysis, we further revealed the molecular mechanism of diterpenoid production and accumulation in different tissues of *S. miltiorrhiza* and *S. grandifolia*. In addition, we identified key transcription factors that regulate tanshinone biosynthesis via direct transcriptional regulation of their pathway gene targets. Furthermore, several key genes involved in diterpene biosynthesis in both species were quantitated, and their function in the regulation of tanshinone biosynthesis in root and leaf tissues of *S. miltiorrhiza* and *S. grandifolia* was investigated. The tissue-specific distribution characteristics of the diterpenoids in *Salvia* were determined, thus providing new insights into the synthetic biology of diterpenoids.

## Results

### Microscopic analysis of cross-sections of *S. miltiorrhiza* and *S. grandifolia*

**Figure 1:**
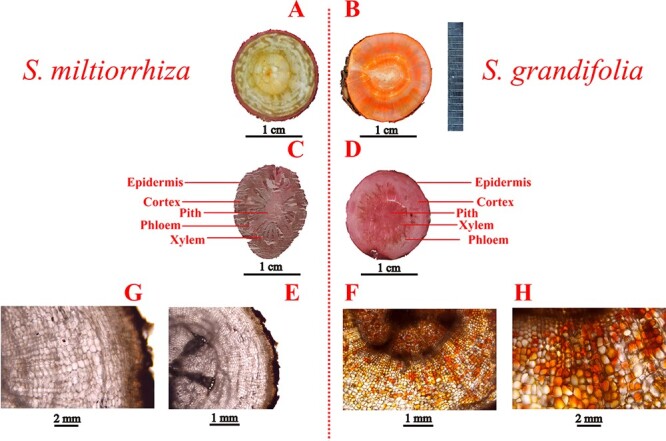
. Primary structure of *S. miltiorrhiza* and *S. grandifolia* roots. (A, B) Cross-sectional optical images of *S. miltiorrhiza* and *S. grandifolia* roots. (C, D) Hematoxylin and eosin staining diagram of *S. miltiorrhiza* and *S. grandifolia*. (E–H) Microscopic characteristics of root cross-sections of *S. miltiorrhiza* and *S. grandifolia* roots . 40×; the eyepiece is 10×, the objective lens is 4×, 100×, the eyepiece is 10×, the objective lens is 10×.

The periderm, xylem, and phloem of *S. grandifolia* roots showed an obvious red color that was similar to the color of tanshinones. To further analyze the color variation of *S. miltiorrhiza* and *S. grandifolia* roots, cross-sections of the roots were subjected to optical, staining, and microscopic analyses (Fig. 1). As shown in Fig. 1, cross-sections of *S. miltiorrhiza* and *S. grandifolia* roots showed anatomical diversity. After hematoxylin and eosin staining, tissues such as the epidermis, cortex, phloem, and xylem were clearly distinguished in the roots of both plants (Fig. 1C and D). In *S. miltiorrhiza*, tissue subdivisions, such as the cortex, phloem, and xylem, were observed to consist mostly of white and yellowish cells, with a distinct dark red color visible only in the periderm of the roots (Fig. 1A). In contrast, *S. grandifolia* sections showed a red color in all regions except the cortex partition (Fig. 1B).

The layered structure of the epidermis, cortex, and vascular cylinder was seen in the cross-sections of *S. miltiorrhiza* roots in the primary growth state. The epidermis consisted of a layer with closely spaced rectangular cells, and the cortical tissue within the epidermis consisted of thin-walled cells. The vascular tube consisted of a layer with circumferential cells, primary xylem and a primary ligament. In the central part of the root there was a pith and a pith cavity, which was large and hollow. All of the cells were white or colorless, except for the epidermal cells, in which a dark red substance was visible (Fig. 1G and E). Notably, a large number of dark red and orange-yellow ‘oil packs’ were observed throughout the microstructure of *S. grandifolia* sections, and the entire cell volume was much larger than the cell volume of *S. miltiorrhiza* at the same magnification (Fig. 1F and H). We observed complete cellular structures in both the xylem and phloem of *S. miltiorrhiza* and *S. grandifolia* but not in the epidermis*,* likely because most of the cells in the root epidermis had died.

### Metabolomic multivariate statistical analysis of *S. miltiorrhiza* and *S. grandifolia*

Comprehensive secondary metabolite profiling of the *S. miltiorrhiza* and *S. grandifolia* roots was performed using an untargeted metabolomics approach. Total ion flow maps of *S. miltiorrhiza* and *S. grandifolia* were obtained by UHPLC-Q-Exactive mass spectrometry (MS) (Supplementary Data Figs S2 and S3). Analyses using in-house tools and other databases systematically identified 76 compounds, which were used to generate metabolomes for *S. miltiorrhiza* and *S. grandifolia* (Supplementary Data Table S1)*.* The majority of the metabolites were tanshinones, phenolic acids, flavonoids, carboxylic acids and terpenoids (Supplementary Data Table S2). The cleavage patterns of the mass spectrometric fragments of salvianolic acid B, dihydrotanshinone I, rosmarinic acid, lithospermic acid, cryptotanshinone, and tanshinone IIA were inferred by combining the chemical bonding patterns of different types of compounds (Supplementary Data Figs S4–S9).

**Figure 2:**
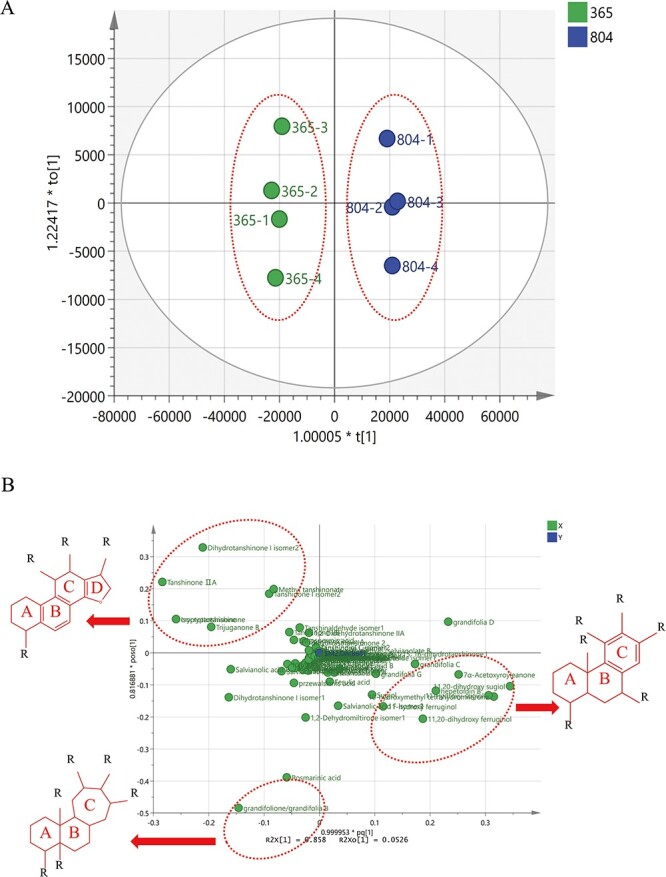
Results obtained from non-targeted metabolomics and chemometric analysis of *S. miltiorrhiza* and *S. grandifolia*. (A) OPLS–DA analysis of *S. miltiorrhiza* and *S. grandifolia* roots. 365, *S. miltiorrhiza*; 804, *S. grandifolia*. (B) S-plot derived from OPLS–DA analysis of *S. miltiorrhiza* and *S. grandifolia* roots.

Supplementary Data Fig. S10 presents a heat map that illustrates the varying abundance of metabolites of different species in the two plants. The relative contents of tanshinone IIA, tanshinone I, and cryptotanshinone were significantly higher in *S. miltiorrhiza* roots than in *S. grandifolia* roots. In particular, the precursor compounds of tanshinone synthesis (11-hydroxy-sugiol, 11,20-dihydroxy-sugiol, and 11,20-dihydroxy-ferruginol) were significantly more abundant in the roots of *S. grandifolia* than in the roots of *S. miltiorrhiza*, but tanshinone analogs were not abundant in *S. grandifolia*. Thus, we speculated that *S. miltiorrhiza* and *S. grandifolia* may have different downstream biosynthetic pathways for tanshinones. The abundance of phenolic acids, such as salvianolic acid B, lithospermic acid, and salvianolic acid G, was also significantly higher in *S. miltiorrhiza* roots than in *S. grandifolia* roots. Grandifolia C, grandifolia D, grandifolia G, and nepetoidin B were significantly more abundant in *S. grandifolia* than in *S. miltiorrhiza*. The abundance of metabolites in the two species was quantified using chemometric methods. Principal component analysis (PCA) and orthogonal partial least squares discriminant analysis (OPLS–DA) clearly distinguished the metabolic differences between the roots of the two species (Fig. 2A, Supplementary Data Fig. S11). The S-plots obtained from OPLS–DA analysis further verified the significant metabolic differences between *S. miltiorrhiza* and *S. grandifolia* (Fig. 2B). The differences between the two species were mainly concentrated in the marginal areas (Fig. 2B). These differential distributed metabolites were mainly tanshinone diterpenoids with a 14,16-ether D-ring, phenolic abietane type diterpenoids with no furan D-ring, or compounds with a unique seven-membered C-ring.

### Optimization of desorption electrospray ionization instruments and selection of spray solvents

Optimization of the key parameters of the desorption electrospray ionization (DESI) system is extremely important for the resolution, sensitivity, and ionization efficiency of the analysis method [24]. Therefore, at the initial stage of the optimization experiment, two homogeneous samples were prepared to identify the key parameters of the system. Slides with special red and black marks and evenly distributed handwriting were used as the samples (Supplementary Data Fig. S12). The responsivity of the mass spectrum signal was taken as the reference standard to debug each parameter, explore the effect of the position of the needle, and obtain the optimal signal under each parameter (Supplementary Data Fig. S13).

**Figure 3:**
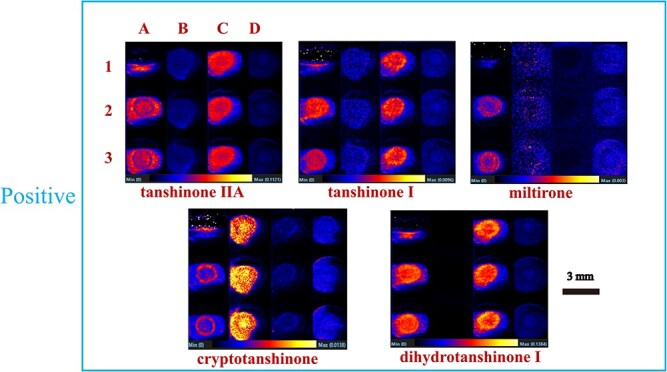
Imaging of *S. miltiorrhiza* standard under positive ions. A, B, C, and D represent 70% methanol, 80% methanol, 80% methanol +0.1% formic acid, 90% methanol +0.1% formic acid respectively; 1, 2, and 3 represent three sample repetitions.

For DESI–MSI, the selection of spray solvents is critical for adequate dissolution of the analyte and the formation of droplets [25, 26]. Therefore, tanshinone and salvianolic acid standards were prepared to explore the effects of 70% MeOH, 80% MeOH, 80% MeOH + 0.1% formic acid and 90% MeOH + 0.1% formic acid spray solvents on the imaging results. As shown in Supplementary Data Fig. S14, the standard *S. miltiorrhiza* is best characterized under DESI–MSI in both positive and negative ion modes. In positive ion mode (Fig. 3, Supplementary Data Fig. S15), 70% MeOH and 80% MeOH + 0.1% formic acid spray solvents gave significantly better imaging response than 80% MeOH and 90% MeOH + 0.1% formic acid spray solvents for tanshinone IIA, tanshinone I, and dihydrotanshinone I. However, only 70% MeOH gave the best imaging response for miltirone. Sample information regarding tanshinones was barely able to be collected using other solvents, and the detection of cryptotanshinone was poor under acidic conditions. In negative ion mode (Supplementary Data Fig. S15), as the proportion of MeOH in the solvent increased, the intensity of signals for salvianolic acid A, salvianolic acid B, salvianolic acid C, ferulic acid, rosmarinic acid, and lithospermic acid decreased, together with the imaging response. Furthermore, we investigated whether acidity affected the adsorption and desorption of the compounds. The resultsshowed that only salvianolic acid B and salvianolic acid C were better visualized under acidic conditions. Acidic conditions had little effect on the imaging effect of salvianolic acid A and lithospermic acid, but it had a significant inhibitory effect on the imaging effect of ferulic acid and rosmarinic acid. Imaging was poor in the MeOH and formic acid mixture at higher MeOH concentrations (90% methanol + 0.1% formic acid).

Therefore, it is feasible to use 70% MeOH as the solvent to image secondary metabolites from the roots of *S. miltiorrhiza* and *S. grandifolia*, as this enables the visualization of the spatial distribution of the most characteristic quality markers on the cross-sectional surface of the roots of these two species. The images obtained using this method reflect information about the compounds on the object’s surface, and lay the foundation for further imaging analysis of plant tissue samples.

### DESI–MSI spatial analysis of diterpenoid biosynthetic pathways in the roots of *S. miltiorrhiza* and *S. grandifolia*

Diterpenoids are the most abundant and well characterized secondary metabolites of the genus *Salvia*. Although important breakthroughs have been made in the investigation of their biosynthetic pathways and the localization of enzymes that catalyze their synthesis, little is known about the spatial distribution of intermediates involved in the biosynthetic pathway of diterpenoids in the root tissues of different *Salvia* species. In this study, the sites of accumulation of the major intermediates involved in the biosynthesis of diterpenoids were visualized by DESI–MSI. The tanshinone and carnosol biosynthetic pathways are important sources of diterpenoid components, and their characterization with imaging techniques revealed the typical structural features and spatial distribution of the intermediates involved in these pathways. The metabolites putatively assigned in *S.miltiorrhiza * and *S. grandifolia* root tissues by DESI MSI (Supplementary Data Table S3). Figure 5 shows the biosynthetic pathways of the diterpenoid components and the ion images of *S. miltiorrhiza* and *S. grandifolia* roots.

**Figure 4:**
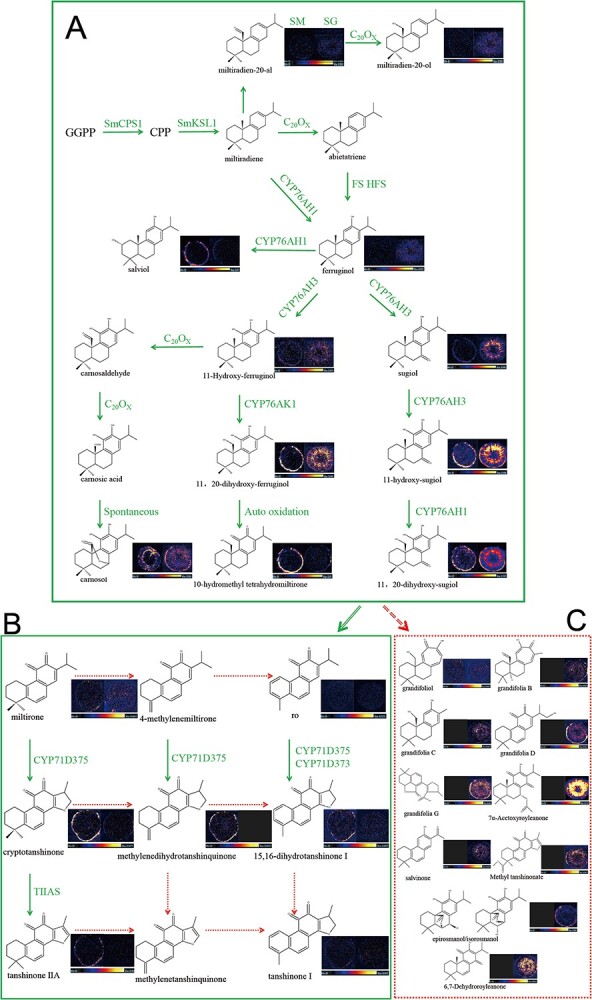
Spatial distribution of diterpenoid biosynthetic pathways in *S. miltiorrhiza* and *S. grandifolia* roots. SM, *S. miltiorrhiza*; SG, *S. grandifolia*.

Thirty-three metabolites involved in the diterpenoid biosynthetic pathway were localized in *S. miltiorrhiza* and *S. grandifolia* roots. These were mainly key secondary metabolites of the carnosol and tanshinone synthesis pathways. As shown in Fig. 4, there were both similarities and differences in the distribution patterns of the target metabolites in the two species. Three upstream precursors (GGPP, CPP, and miltiradiene) could not be detected using the metabolomics approach as they were present in only trace concentrations (Fig. 4A). The tricyclic diterpenes in the tanshinone synthetic pathway, such as 11-hydroxy-sugiol, 11-hydroxy-ferruginol, 11,20-dihydroxy-sugiol, and 11,20-dihydroxy-ferruginol, were widely distributed in the xylem, phloem, and periderm of *S. grandifolia*. These compounds were specifically distributed in the pericardium of *S. miltiorrhiza* and at much lower relative concentrations than in the pericardium of *S. grandifolia*, which is consistent with the metabolomic quantification results. Carnosol was mainly distributed in the periderm and phloem of *S. miltiorrhiza*, but in the periderm of *S. grandifolia*.

In downstream of the tanshinone synthetic pathway, miltirone is transformed via a series of enzymes to form a 14,16-ether D-ring-containing heterocycle, which differs from the other diterpenoid components. Further oxidation of this heterocycle typically results in the formation of dihydrofuran-tanshinone and furan-tanshinone. Tanshinones containing a furan ring were mainly concentrated in the periderm of *S. miltiorrhiza*, and were not present in significantly high concentrations in the cortex or xylem. In comparison, these naturally occurring compounds with rich medicinal value were not clearly detectable in *S. grandifolia*. For example, tanshinone IIA, a diterpenoid component of tanshinone has anticancer, anti-inflammatory, and cardiovascular protective effects [27–29], and precisely localizes near the epidermis of target tissues, but our results showed no significant localization in *S. grandifolia* tissues. The spatial distribution of phenolic diterpenoids and furan cyclic diterpenoids for the entire biosynthetic pathway in *S. miltiorrhiza* remained consistent in the epidermis, as verified by the red coloration of the epidermis in the roots of *S. miltiorrhiza*. Interestingly, the upstream intermediates of the tanshinone synthesis pathway were significantly concentrated in the xylem, phloem, and periderm of *S. grandifolia*, while the final products of the pathway did not significantly concentrate in roots of *S. grandifolia* (Fig. 4B). This shows that the tanshinone biosynthetic pathway is active in both species: *S. grandifolia* mainly contained intermediate metabolites of the upstream pathway, while *S. miltiorrhiza* mainly contained intermediate metabolites of the downstream pathway. Therefore, metabolic pathway branching clearly occurs at the point where the furan D-ring is formed, and the key metabolites in the upstream biosynthetic pathway of *S. grandifolia* are not used only for the biosynthesis of tanshinones.

We sought to determine whether there were other branches in diterpenoid biosynthetic pathways in *S. grandifolia.* We determined the locations of accumulation of five compounds and five diterpenoid components in *S. grandifolia*. These components accumulated in large quantities in *S. grandifolia.* Grandifoliol (a light yellow powder) was widely distributed in the root, whereas the distribution of grandifolia C (white amorphous powder), grandifolia D (red amorphous powder), grandifolia B (yellow crystals), grandifolia G (white amorphous powder), salvinone (red amorphous powder), methyltanshinonate (red amorphous powder), and 6,7-dehydroroyleanone/taxodione (red amorphous powder) were perfectly consistent with the spatial distribution of the red coloration in the epidermis and xylem of *S. grandifolia.* Of these compounds, grandifoliol and grandifolia B, isolated from *S. grandifolia*, contain unique seven-membered rings, and none of the other compounds had a furan ring similar to tanshinone. Based on these results and the tissue localization images, we speculated that there may be a branch from A to C in the diterpenoid biosynthetic pathway (Fig. 4C).

**Figure 5:**
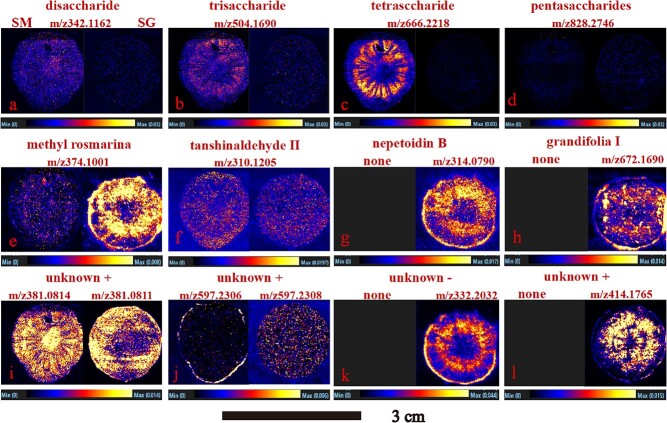
Imaging of other functional metabolites in roots of *S. miltiorrhiza* and *S. grandifolia*. SM, *S. miltiorrhiza*; SG, *S. grandifolia*.

**Figure 6:**
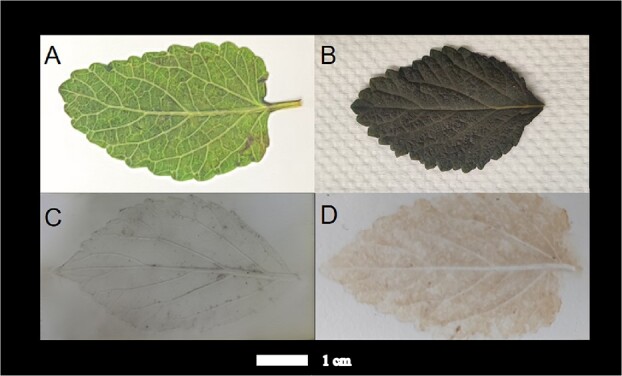
Effects of four treatments of *Salvia* leaves. (A) Untreated fresh leaf sample. (B) Chloroform treatment. (C) TLC imprinting. (D) Teflon-imprinted.

### Spatial dynamics of other functional metabolites in the roots of *S. miltiorrhiza* and *S. grandifolia*

In the roots of *S. miltiorrhiza* and *S. grandifolia*, the majority of non-diterpenoid metabolites, such as phenolic acids and carbohydrates, were also detected in both negative ion and positive ion modes. The tissue distributions of ions of components such as phenolic acids and polysaccharides were visualized in the roots of the two species (Fig. 5). In recent years, polysaccharides isolated from *S. miltiorrhiza* have attracted increasing attention due to their significant antitumor, antioxidant, antiproliferative, and immunomodulatory activities and their ability to improve the immune response [30, 31]. Compared with the polysaccharides in *S. grandifolia*, those in *S. miltiorrhiza* had more obvious tissue specificity. For example, disaccharides, trisaccharides, and tetrasaccharides were found to be accumulated primarily in the xylem ray regions of *S. miltiorrhiza* (Fig. 5a–d). These polysaccharides may be stored in the xylem to supply energy to the surrounding tissues. After image normalization, it was found that there was a higher concentration of tetrasaccharides than other polysaccharides, indicating that tetrasaccharides are one of the main polysaccharide components in *S. miltiorrhiza* [32]. In addition, methyl rosmarinate was mainly distributed in the epidermis and xylem of *S. miltiorrhiza*, while it was distributed in all parts of *S. grandifolia*. Tanshinaldehyde II was dispersed within various tissues in both species (Fig. 5e and f). In *S. grandifolia*, nepetoidin B and grandifolia I were distributed in the epidermis and xylem (Fig. 5g and h). Therefore, there were relatively few functional metabolites in the cortex, which plays a role in conduction and transport of compounds throughout biological tissues. In addition, some metabolite ions showed region-specific distributions, which could be used to clearly characterize the heterogeneity of *S. miltiorrhiza* and *S. grandifolia* roots (Fig. 5i–l).

### Spatial distribution of metabolites in the leaves of *S. miltiorrhiza* and *S. grandifolia*

To further understand the similarities in secondary metabolite distributions in two plants, we focused on the characteristics of metabolite accumulation and distribution in the leaves of the two species*.* The results of the four treatments are shown in Fig. 6. Untreated leaf epidermal cells and leaves with residual wax had an obvious impenetrable physical barrier that blocked passage of the charged microdroplets of the extraction solvents (Fig. 6A). Surface pretreatment with chloroform was expected to improve the signal intensity, but the experimental results showed this organic solvent caused permanent damage to fresh leaves (Fig. 6B). Leaves treated with thin-layer chromatography (TLC) aluminum foil were also unsuitable as experimental material due to the diffusion of metabolites and the low concentrations of imprinted metabolites (Fig. 6C). Therefore, polytetrafluoroethylene (PTFE)-imprinted leaves were used for detecting the distribution of metabolites in the target leaves (Fig. 6D).

**Figure 7:**
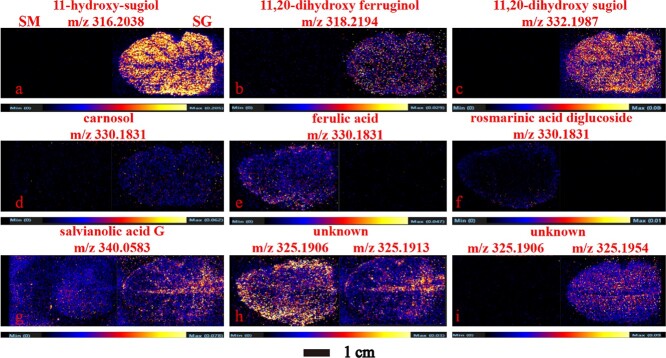
Imaging of representative metabolites in *S. miltiorrhiza and S. grandifolia* leaves. SM, *S. miltiorrhiza*; SG, *S. grandifolia*.

**Figure 8:**
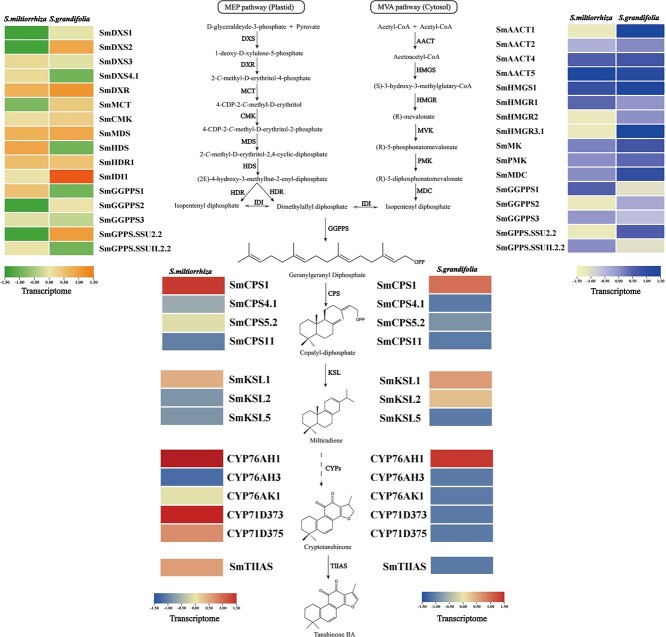
Gene expression heat maps of the tanshinone pathways in *S. miltiorrhiza* and *S. grandifolia.*

Previous work has shown that there are a significant proportion of intermediates of the upstream section of the tanshinone biosynthetic pathway in *S. grandifolia* leaves, and they are widely distributed, which indicates that *S. grandifolia* leaves are a potential tanshinone production organ (Fig. 7a–c). The demand for carnosol, a product of the carnosol biosynthetic pathway, is increasing in the food, nutrition, health, and cosmetics industries [33]. In this study, carnosol was found to be distributed in almost all tissues of the leaves of *S. grandifolia*, which provides the basis for further studies of the homology of *S. grandifolia* in the fields of medicine and food science (Fig. 7d). Ferulic acid is a type of organic acid that is widely distributed in the plant kingdom and has received much attention for its important biological functions and applications. Previous studies have shown that ferulic acid is an important free radical scavenger and it has a protective effect on the skin [34]. In this study, ferulic acid was widespread in whole leaves of *S. miltiorrhiza*, especially in the leaf margins, which may provide new perspectives on the biosynthesis of ferulic acid in the leaves of *S. miltiorrhiza* (Fig. 7e). The glycoconjugates of rosmarinic acid were concentrated and distributed only at the leaf margins in *S. miltiorrhiza* (Fig. 7f). Unexpectedly, the concentration of salvianolic acid G was higher in the leaves of *S. grandifolia* than in those of *S. miltiorrhiza*, and it was mainly located around the leaf veins. This finding may guide the further exploration of medicinal parts of *S. grandifolia* (Fig. 7g)*.* In addition, as shown in Fig. 8h and i, we found a large number of metabolites with specific distribution patterns, which lays the foundation for the discovery of new compounds.

### Transcriptomics analysis of *S. miltiorrhiza* and *S. grandifolia* roots revealed the differential mechanism of secondary metabolism

In order to further reveal the molecular mechanisms of terpenoid-related accumulation differences between *S. grandifolia* and *S. miltiorrhiza,* RNA-seq data were obtained from roots of both species. A total of 24 587 785 sequence reads from *S. miltiorrhiza* and 24 406 987 sequence reads from *S. grandifolia* were obtained. Compared with *S. miltiorrhiza*, 69 969 sequences were upregulated and 62 807 sequences were downregulated in *S. grandifolia* (Supplementary Data Table S4). A total of 132 776 differentially expressed genes (DEGs) were annotated into more than 20 metabolic pathways (Supplementary Data Figure S16, Supplementary Data Table S5). The 9198 differentially generated genes were divided into 20 significantly enriched KEGG (Kyoto Encyclopedia of Genes and Genomes) pathways.

The expression levels of key genes for tanshinone biosynthesis in the two plants were further analyzed. As shown in Fig. 8, the expression levels of key genes in the MEP pathway (Methyl-D-Erythritol Phosphate Pathway) in *S. grandifolia* roots were significantly higher than those in *S. miltiorrhiza* roots*.* For example, the expression levels of *SmDXR*, *SmMCT*, and *SmIDI1* in the root of *S. grandifolia* were ~1.66, 10.59, and 36.53 times those in the root of *S. miltiorrhiza*, respectively. Meanwhile, in the MVA (Mevalonate Pathway) pathway, *SmAACT1*, *SmHMGS1*, *SmMK*, and *SmMDC* were also highly expressed in *S. grandifolia*, with expression levels ~1067.20, 7.97, 5.97 and 12.78 times those of *S. miltiorrhiza*, respectively*.* In addition, the expression levels of the upstream pathway genes in tanshinone biosynthesis, such as *SmGPPS.SSU2.2*, *SmKSL1*, and *SmKSL2*, were also significantly higher in *S. grandifolia*: they were ~48.88, 2.52, and 24.85 times the levels in *S. miltiorrhiza*, respectively. In contrast, the downstream pathway genes in tanshinone biosynthesis, such as *CYP76AK1*, *CYP76AH1*, *CYP71D375*, and *TIIAS*, were highly accumulated in *S. miltiorrhiza*: they were ~7.02, 1.41, 35.82, and 26.86 times the levels in *S. grandifolia*. The findings are in agreement with the accumulation of metabolites from both *S. grandifolia* and *S. miltiorrhiza*.

CYP450 mono-oxygenases are multifunctional biocatalysts, which play a crucial role in the formation of the parent nucleus and post-modification reactions of terpenoids [35, 36]. In our study, we identified 271 CYP450 genes that were differentially expressed between *S. miltiorrhiza* and *S. grandifolia*, including CYP genes involved in tanshinone biosynthesis, such as *SmCYP76AH1*, *SmCYP76AH3*, *SmCYP76AK1*, *SmCYP71D373*, and *SmCYP71D375* [16–18] (Supplementary Data Table S7). Numerous studies have shown that transcription factors such as MYB, bHLH, and WRKY are involved in regulating the accumulation of terpenoids, especially tanshinones [37–39]. We also identified a large number of differentially expressed transcription factor genes in both species, including 168 MYB, 47 bHLH, and 176 WRKY (Supplementary Data Tables S8–S10). These DEGs may be related to the differences in diterpenoid accumulation between the two plants.

### Expression analysis of key genes involved in diterpenoid biosynthesis in *S. miltiorrhiza* and *S. grandifolia*

**Figure 9:**
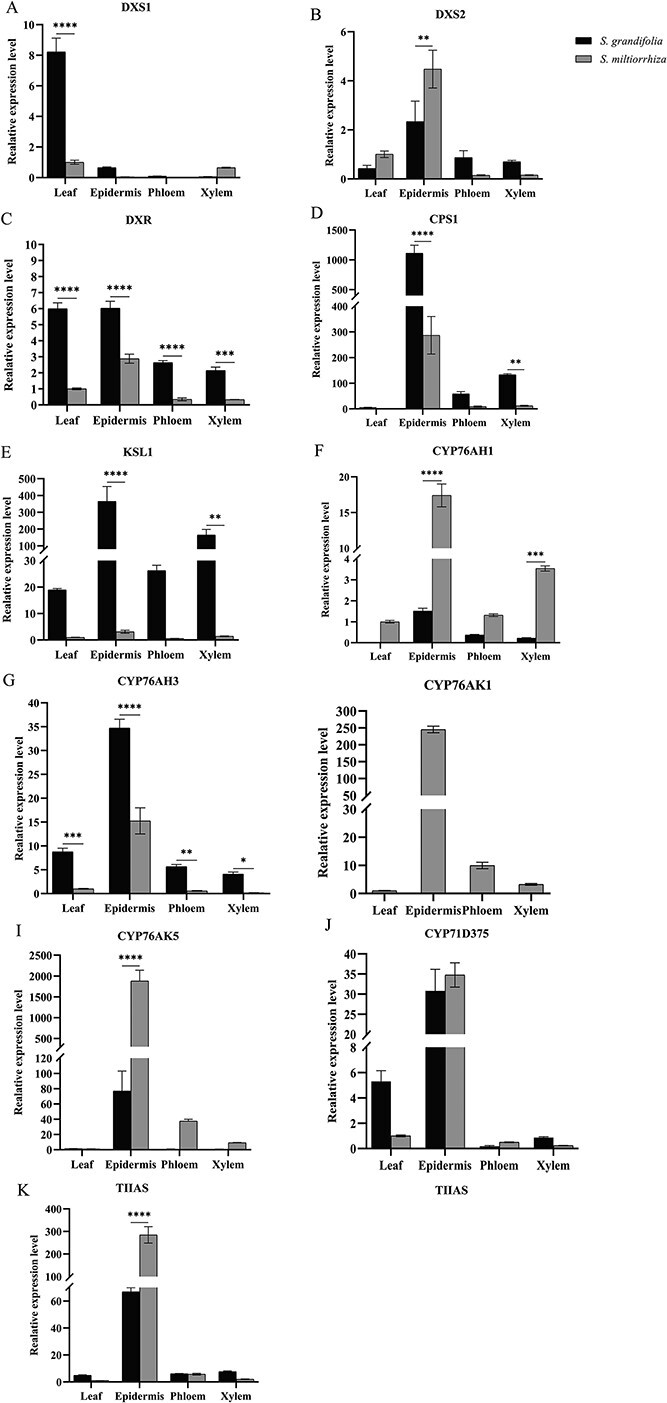
Expression levels of the 12 candidate genes by FPKM in roots of *S. grandifolia* and *Salvia miltiorrhiza*. ^*^P < .05, ^**^P < .01, ^***^P < .001, ^****^P < .0001.

To further explain the mechanisms of the distribution of diterpenoids in different parts of *S. grandifolia* and *S. miltiorrhiza*, we simultaneously analyzed the tissue-specific expression patterns of key genes involved in diterpene biosynthesis in both species using RT–qPCR (Fig. 9). The results showed that *DXS2*, *DXR*, *CPS1*, *KSL1*, *CYP76AK1*, *CYP76AH1*, *CYP76AH3*, *CYP76AK5*, *CYP71D375*, and *TIIAS*, key genes of the tanshinone biosynthetic pathway, were significantly highly expressed in the root periderm of *S. miltiorrhiza*. All the pathway genes showed low expression in the xylem and phloem of *S. miltiorrhiza* roots, which were consistent with the observation that tanshinones were primarily distributed in the root epidermis.

In *S. grandifolia*, the genes encoding the downstream pathway of tanshinone biosynthesis, such as *CYP76AH1*, *CYP76AH3*, *CYP76AK5*, *CYP71D375*, and *TIIAS*, were also significantly upregulated in the periderm. In particular, the expression of *CYP76AK5* and *TIIAS* in the epidermis was 60-fold higher than that in other parts, which was consistent with the result that the tanshinones were mainly distributed in the periderm. Genes such as *DXS2*, *DXR*, and *KSL1* were also highly expressed in the phloem and xylem, which also suggested that the high contents of intermediate metabolites such as 11-hydroxy-sugiol in the xylem and phloem of *S. grandifolia* roots might be related to these genes. *DXS1* and *KSL1* were significantly highly expressed in the leaves of *S. grandifolia*, and it was speculated that they may be involved in the accumulation of 11-hydroxy-sugiol, 11,20-dihydroxy-sugiol and 11,20-dihydroxy-ferruginol in the leaves.

Compared with *S. miltiorrhiza*, the expressions of diterpenoid biosynthesis upstream pathway genes such as *DXR*, *CPS1*, *KSL1*, and *CYP76AH3* were significantly higher in *S. grandifolia*, while the expressions of tanshinone biosynthesis downstream pathway genes such as *CYP76AK1*, *CYP76AK5*, *CYP71D375*, and *TIIAS* were lower. This is consistent with the results of higher accumulation of upstream metabolites of diterpenoid biosynthesis in *S. grandifolia* and higher content of downstream metabolites in *S. miltiorrhiza*. It is suggested that the high expression of genes in the upstream pathway of diterpenoid biosynthesis in *S. grandifolia* is likely to lead to the high accumulation of intermediate metabolites such as 11-hydroxy-sugiol, 11,20-dihydroxy-sugiol and 11,20-dihydroxy-ferruginol, and the high expression of *CYP76AK1*, *CYP76AK5*, *CYP71D375*, and *TIIAS* is a significant influential factor for the high-level accumulation of tanshinones in *S. miltiorrhiza*.

## Discussion

Terpenoids are hydrocarbons that are widely found in plants and are the largest class of naturally occurring products [40]. The 6/6/6 tricyclic diterpenes and 6/6/6/5 furan cyclotetracyclic diterpenes of the tanshinone synthesis pathway are widely distributed in *Salvia* species [41, 42]. The tricyclic diterpene components, such as carnosic acid, are the main components of European medicinal *Salvia* species, while the tetracyclic diterpene quinones with a furan ring, such as tanshinones, are abundant in East Asian *Salvia* species [43]. *Salvia grandifolia* is mainly distributed in East Asian, but it is more closely related to European *Salvia* species than other East Asian species [44, 45]. We found that the metabolites of *S. grandifolia* were very similar to those of European *Salvia* species, as high concentrations of ABC cycloditerpenoids were present in the roots of *S. grandifolia*, and these concentrations were significantly higher than in other East Asian *Salvia* species, as represented by *S. miltiorrhiza*. However, a low concentration of tetracyclic tanshinones was also present. In terms of secondary metabolite profiles, the results showed that *S. grandifolia* was more similar to European *Salvia* species than East Asian *Salvia* species, but it retained the distribution characteristics of secondary metabolites seen in some East Asian *Salvia* species. This finding indicates that the evolution of secondary metabolites is largely consistent with species evolution. The 6/6/7 carbocyclic skeleton tricyclic diterpenoids have rarely been reported in *Salvia,* but przewalskin A has been detected in *Salvia przewalskii* Maxim. [46], salyunnanin A has been detected in *Salvia yunnanensis* C.H.Wright [47] and hassanane has been detected in *Salvia apiana* Jeps. [48]. We also found there was a wide distribution of 6/6/7 cyclic diterpenoids with heptacyclic C rings in *S. grandifolia*, and provided evidence of the biosynthetic mechanism of diterpenoids of this structural type.

The periderm of *S. miltiorrhiza* is the primary distribution site for tanshinone IIA and cryptotanshinone, as demonstrated by multiple studies [49], and a method based on matrix-assisted laser desorption/ionization MSI also confirmed that tanshinone components are mainly concentrated in the periderm, but their presence in other parts of the plant has not been reported [50, 51]. We demonstrated by DESI–MSI that tanshinone diterpenoids with a 14,16-ether D-ring were mainly distributed in the root periderm of *S. miltiorrhiza*, indicating that the periderm of *S. miltiorrhiza* roots is the main site of tanshinone metabolite storage. It has been reported that the key genes for the biosynthesis of tanshinone components were highly expressed mainly in the periderm, but expressed at lower levels in the xylem and phloem [52]. Our results also revealed that the genes *CPS1*, *KAL1*, *CYP76AH1*, *CYP76AK1*, *CYP76AK5*, and *TIIAS* were more highly expressed in the root periderm of *S. miltiorrhiza*, while expression was lower in the root xylem and phloem as well as in the leaves, which may be an important reason for the distribution of salvinone components mainly in the root periderm area. The color of the whole root of *S. grandifolia* was very similar to the color of tanshinone, and MSI revealed that tanshinones were only present in low concentrations in the periderm. In *S. grandifolia*, we also found high expression of key genes of the downstream pathway of tanshinone component synthesis in the periderm, which suggested that the biosynthesis mechanism of tanshinone components in *S. miltiorrhiza* and *S. grandifolia* is similar, both of them being synthesized and accumulated mainly in the periapical part of the root. The distribution patterns of tanshinone diterpenoids were the same in the two species, indicating that the diterpenoids containing a furan tetracycline were specifically distributed in the root pericardium of *S. miltiorrhiza* and *S. grandifolia.* However, tricyclic diterpenoids from the upstream section of the tanshinone synthesis pathway accumulated in high concentrations in the periderm, xylem, and phloem of *S. grandifolia*, while in *S. miltiorrhiza* they were only located in the periderm. *DXS2*, *DXR*, and *KSL1* are key genes in the upstream pathway of diterpenoid biosynthesis, and we detected high expression of these genes in both xylem and phloem of *S. grandifolia* root. It is hypothesized that these genes may be involved in the accumulation of intermediate metabolites such as 11-hydroxy-sugiol in xylem and phloem, resulting in the high accumulation of these components in xylem and phloem. The transport of secondary metabolites in plants is essential for the maintenance of cell growth, development, survival, homeostasis, and the coordination of their entire biochemical system [53]. The metabolites of the entire tanshinone biosynthetic pathway were only located in the periderm of *S. miltiorrhiza*, while in *S. grandifolia* all regions of the root except the cortex contained metabolites involved in tanshinone biosynthesis. Tanshinone diterpenoids have been reported to regulate the composition of the root microbiome [54], based on the fact that tanshinones are in direct contact with the soil in the outermost layer of the root system. Therefore, the distribution pattern of tanshinones is closely related to soil microbes and they may have antagonistic or symbiotic effects on specific microbial communities in the soil. In addition, we detected many diterpenoids involved in the upstream section of the tanshinone biosynthesis pathway in *S. grandifolia* leaves. Thus, tissue culture of *S. grandifolia* leaves may serve as a good resource for the industrial scale biosynthesis of tanshinone, and facilitate the application of synthetic biology with plant tissues as carriers. The identification of these metabolites in different parts of plants is helpful for delineating the sites of their biosynthesis and the mechanisms of tanshinone transport.

Focusing on the spatial variation of metabolites associated with metabolic pathways is another approach to the study of changes in plant physiology. Therefore, exploring the spatial variation of metabolites will increase our understanding of secondary metabolic mechanisms. There is a dependency on both the mevalonate pathway in the cytoplasm and the methyl erythritol phosphate pathway in plastids for the production of tanshinones, and there is an exchange of material and information between these two pathways [55–57]. In the tanshinone biosynthesis pathway, GGPP is enzymatically transformed by carbamoyl phosphate synthetase (CPS) to generate copolymer diphosphate (CPP), which is enzymatically transformed by kaunene synthase (KSL) to form the tanshinone precursor, miltiradiene [58]. Miltiradiene can be converted into ferruginol by the CYP family protein, SmCYP76AH1, through catalysis [17], SmCYP76AH3 then hydroxylates the C-7 and/or C-11 positions of ferruginol to produce 11-hydroxy-ferruginol, sugiol and 11-hydroxyl sugiol; the hydroxylation of the C-20 of 11-hydroxyferruginol and 11-hydroxyl sugiol by SmCYP76AK1 produces 11,20-dihydroxy-ferruginol and 11,20-hydroxyl sugiol, and 11,20-hydroxyl sugiol is autoxidized to 10-hydroxymethyl tetrahydromiltirone [18]. Finally, other CYP450 oxidases, decarboxylases, and reductases catalyze the formation of active components, such as tanshinone IIA and cryptotanshinone [59]. There was no pronounced accumulation of final metabolites of the tanshinone biosynthesis pathway in *S. grandifolia* roots. However, many abietane-type diterpenoids from early portions of the biosynthetic pathway that are intermediates in the upstream processes of tanshinone synthesis were detected in *S. grandifolia* roots. There was significantly less of this accumulation of tanshinones in the middle and downstream sections of the tanshinone biosynthetic pathway in *S. grandifolia* than in *S. miltiorrhiza*. This difference clearly occurred when the furan D-ring of tanshinone was formed during tanshinone biosynthesis, which is a step that differentiated the two species. The emergence of such metabolically divergent points may be an evolutionary difference between the two species to adapt to the environment.

### Conclusions

In the present study, multi-omics techniques and DESI–MSI were performed to achieve a comprehensive understanding of the spatial distribution and molecular mechanisms of terpenoid biosynthesis in *S. miltiorrhiza* and *S. grandifolia*. Diterpenoids from *S. miltiorrhiza* were found to be tetracyclic diterpene quinones containing a furan D-ring, while those from *S. grandifolia* were typically phenolic abietane-type tricyclic diterpenoids with a six- or seven-membered C-ring. Tetracyclic diterpenoids were enriched in the periderm of *S. miltiorrhiza* and *S. grandifolia* roots*,* while tricyclic diterpenoids were widely distributed in the xylem, phloem, and periderm of *S. grandifolia* roots. The xylem, phloem, and periderm of *S. grandifolia* roots were enriched in precursors of tanshinone biosynthesis, while the periderm of *S. miltiorrhiza* roots mainly contained the end products of downstream pathways. Metabolic pathway branching clearly occurs in both species when the furan D-ring is forming. The leaves of *S. grandifolia* contained a lot of phenolic tricyclic abietane-type diterpenoids, while the leaves of *S. miltiorrhiza* mainly contained phenolic acids. Genes in the upstream pathway of tanshinone biosynthesis are highly expressed in the root of *S. grandifolia*, and genes in the downstream pathway are highly expressed in the root of *S. miltiorrhiza.* The results of this study will improve our understanding of precise localizations and biosynthesis of terpenoids in *S. miltiorrhiza* and *S. grandifolia*. Combination of multi-omics and DESI–MSI will provide an efficient approach to visualizing the accumulation rules of natural products and provide more meaningful and unique insights into secondary metabolism.

## Materials and methods

### Reagents and materials

Thermo Fisher Scientific (Waltham, MA, USA) enabled the acquisition of ethanol, formic acid, acetonitrile, and ethanol, supplying the necessary reagents. All other reagents were of a superior analytical quality grade. *Salvia miltiorrhiza* was harvested from Yangzhou, Jiangsu Province, China, while *S. grandifolia *was collected from Shanghai National Forest Germplasm Resource Center of Lamiaceae Plant.

### Sample preparation for DESI–MSI experiments

The *S. miltiorrhiza* and *S. grandifolia* samples were washed, and then the taproot and lateral roots were separated. A section of ~1.0–1.5 cm was cut from the middle of a taproot and then immediately immersed in liquid nitrogen to cool for 20 seconds, and subsequently frozen rapidly at −80°C. A freezing microtome at −20°C was used to cut consecutive 10-μm-thick sections from the frozen samples. The tissue sections to be analyzed were removed by adsorption to a glass slide, which was then immediately placed in a vacuum dryer in the dark for 20 minutes. Slides with tissue sections were then scanned using a CanoScan LiDE 400 imaging system (Canon Inc., Tokyo, Japan). The resulting images were used to obtain location information for the tissue sections, after which the slides were placed on a preleveled platform for fixation.

*Salvia miltiorrhiza* and *S. grandifolia* leaves are densely covered with glandular hairs [63], and thus to optimize screening tissue sections from four different types of leaf samples were prepared: untreated leaves [64, 65]; leaves leached with chloroform for wax removal [66, 67]; TLC blots [68–70]; and porous PTFE imprints [71–72]. The leaves of the untreated group were washed only with purified water, to remove the sludge from the surface. The residual water was removed by gently wiping the leaves with absorbent paper, and they were then affixed to the slides using double-sided adhesive tape and subjected to direct imaging. The leaves in the chloroform treatment group were soaked in chloroform for 30 seconds and rinsed with purified water, and then removed and pasted onto glass slides. Leaves in the TLC imprinting group were stretched flat and placed on an aluminum TLC plate. A piece of air-laid paper was gently placed on top of the leaves on the plate, and the resulting paper–leaf–plate combination was sandwiched successively between two pieces of silicone rubber and two aluminum plates. This set-up was compressed with pliers for 30 minutes to form a TLC imprint. Leaves in the PTFE imprinting group were placed on porous PTFE material that was pre-cut to the size of the glass slides. A piece of silicone rubber was attached to an aluminum plate and placed on top of the air-laid paper and an imprint was made using pliers for 30 minutes. Imprinted leaf metabolites were thereby immobilized within the pores of the PTFE.

### LC–MS/MS analysis

Roughly 0.02 g of root tissue was homogenized with 1 ml of MeOH/H_2_O (70:30, v/v), then spun for 5 minutes and exposed to ultrasonic treatment for 20 minutes. After centrifugation at 10 000 rpm for 10 minutes, the sample was passed through a 0.22-μm pore size membrane, and the supernatant was then filled with the filtrate for the LC–MS/MS experiment.

An Accucore C18 column (2.6 μm, 2.1 mm × 5.0 mm; Thermo Fisher Scientific) enabled chromatographic separation to be attained. Temperature and flow rate were adjusted to 40°C and 0.3 ml min^−1^, respectively, for the column. The chromatographic column’s injection volume and detection wavelength were established at 10 μl and 254 nm, respectively. A gradient elution program of 96% A (0–1 minute), 96–60% A (1–43 minutes), 60–5% A (43–53 minutes), 5% A (53–58 minutes), 5–96% A (58–60 minutes) and 96% A (60–63 minutes) was employed using a mobile phase composed of 0.1% formic acid (A) and acetonitrile (B).

Mass spectrometry detection was operated in the positive and negative ion spray modes, and a quadrupole electrostatic field orbital well high-resolution mass spectrometer Q-Exactive detector was used. Parameters were as follows: ion source, ESI (±); spray voltage, 3.0 kV; evaporation temperature, 320°C; sheath gas, 35Arb; auxiliary gas, 8Arb; capillary temperature, 350°C; S-lens RF, 50. For simultaneous collection of positive and negative ions, first-level full scan parameters were: resolution, 70000; AGC target, 3e6; maximum TT, 100 ms; scanning range, 110–1650 m/z. Level 2 data dependency scan (full MS/dd MS2) parameters were: resolution, 17500; AGC target, 1e5; maximum TT, 50 ms; loop count, 5; NCE, 20, 30, 40ev. All measurement data were collected by Aria 1.6.3 and Xcalibur 2.1 software (Thermo Fisher, USA).

### DESI–MSI analysis

All DESI–MSI experiments were performed using a high-resolution Synapt XS HDMS 4 k mass detector (Waters Corporation, Milford, MA, USA). A DESI ion source (Waters Corporation) was placed at the entrance of the mass spectrometer to sample the tissue sections. In full scan mode, both positive and negative ion detection modes were utilized to acquire mass spectrometry data. At a resolution of 20 000, all mass spectra were obtained over an interval of 50–1200 *m*/*z*.

To optimize DESI–MSI, we performed the following actions: (1) taking the slides with special red and black marks and evenly distributed handwriting as samples, and using the responsiveness of the mass spectrometry signal as a reference standard, we debugged each parameter, discussed the influence of needle position, and obtained the optimal signal under each parameter; (2) preparation of tanshinone and salvianolic acid standards, and discussion of the influence of 70% MeOH, 80% MeOH, 80% MeOH + 0.1% formic acid, and 90% MeOH + 0.1% formic acid spray solvent on imaging results.

To obtain good signal intensity, the following DESI parameters were optimized: ES voltage at 3 kV, solvent flow rate at 2 μl/minute, DESI gas pressure at 0.35 MPa, source temperature at 120°C, sampling cone voltage at 40 V, spray impact angle at 75°, emitter tip to surface capillary orifice at 2 mm, emitter tip to ion transfer capillary orifice at 6 mm, ion transfer capillary orifice to surface at 0.5 mm, emitter exposure from sprayer tip at 0.5 mm, and scanning time at 1 second; 70% MeOH as DESI–MSI spray solvent.

### RNA extraction, Illumina sequencing, and transcriptome data analysis

RNA samples were isolated using the RNAprep Pure Plant Plus Kit (#DP441; Tiangen, http://www.tiangen.com). A total of 1.5 μg RNA, generated from each sample, was utilized to create a sequencing library with the NEBNext^®^ Ultra™ RNA Library Preparation Kit (NEB, USA). Sequencing of the library on the Illumina Hiseq 4000 platform yielded dual-ended raw reads of 150 bp. Reads of *S. miltiorrhiza* and *S. grandifolia* were *de novo* assembled using Trinity under default parameters. We took the absolute log_2_ [FPKM (fragments per kilobase of transcript per million mapped reads)] ≥ 1 and the *P*-value of <.05 as the threshold to identify DEGs. DEGs were identified using a threshold of absolute log_2_ (FPKM) ≥1 and *P*-value <.05. By utilizing KEGG (http://www.genome.jp/kegg), a pathway analysis was conducted to elucidate the essential pathways of DEGs.

### Quantitative reverse transcription PCR analysis

Four plant parts (leaf, epidermis, phloem, xylem) of *S. miltiorrhiza* and *S. grandifolia* were immediately collected and frozen in liquid nitrogen. The isolated RNA was reverse transcribed using the FastPure^®^ Universal Plant Total RNA Isolation Kit (Vazyme, RC411-01). RT–qPCR was executed with TB Green^®^ Premix Ex Taq™ Tli RNaseH Plus (TAKARA, RR420A). Supplementary Data Table S6 displays the relative expression of CYP76K1 (*S. miltiorrhiza*) when SmActin and SgActin are employed as gene-specific primers for internal reference. The expression levels of CYP76AK6 (*S. grandifolia*), CYP76AK5, KSL1, DXS1, DXS2, CPS1, CYP76AH1, CYP76AH3, DXR, CYP71D375, and TIIAS were calculated using the formula 2−ΔΔCt.

### Data analysis

DESI imaging was performed using HDImaging v1.5 software (Waters Corporation) for high-resolution imaging reproduction and the images were reconstructed using linear smoothing. The secondary mass spectrometry data, online databases (e.g. SciFinder, Massbank, and PubMed), and previously published research were utilized to identify the metabolites and their potential fragmentation pattern. To assess overall metabolic differences between sample regions and determine the magnitude of variation between samples within groups, unsupervised PCA was conducted using SIMCA-P 14.1 (Umetrics Corporation, Umea, Sweden) software. To obtain information about metabolites that were significantly different between subgroups, supervised OPLS–DA was performed.

## Acknowledgements 

This work was financially supported by Zhejiang Provincial Natural Science Foundation of China (No. LR21H280002), a Key Scientific and Technological Grant of Zhejiang for Breeding New Agricultural Varieties (No. 2021C02074), the Key project of the Central Government: Capacity Building of Sustainable Utilization of Traditional Chinese Medicine Resources (No. 2060302), and the National Natural Science Foundation of China for State Key Laboratory (No. 81973415).

## Author contributions

J.X. and G.G.L. were tasked with the experimental study, data analysis, and manuscript composition; L.Z. was responsible for qRT–PCR; Y.B.H., J.Y., and J.G. for sample collection; and Z.C.Q., Z.H.L., G.L.Z., S.C.X., and X.J.S. for composition testing. Authors X.D.Z., Y.K.W., Z.S.L., and D.F.Y. were tasked with the design, preparation, and revision of the manuscript in its entirety.

## Data availability

All relevant data are available within the article and its supplementary materials.

## Conflict of interest

The authors declare that they have no conflicts of interest.

## Supplementary data

Supplementary data are available at *Horticulture Research* online.

## Supplementary Material

Web_Material_uhad109
