# Supplementary material for: Unveiling the spatial distribution and molecular mechanisms of terpenoid biosynthesis in Salvia miltiorrhiza and S. grandifolia using multi-omics and DESI–MSI
Source: Hortic Res. 2023 May 31;10(7):uhad109. doi: 10.1093/hr/uhad109 (PMC10419090; doi:10.1093/hr/uhad109)
Supplement: Web_Material_uhad109 [file web_material_uhad109.zip › Supplementary Material.doc]

**Supplementary material**

**Unveiling the spatial distribution and molecular mechanisms of terpenoids biosynthesis in *Salvia miltiorrhiza* and *S. grandifolia* using untargeted metabolomics and DESI-MSI**

**Table of Content**

| **Table/Figure** | **Title** | **Page** |
| --- | --- | --- |
| **Fig. S1** | The crude drug of *S. miltiorrhiza* and *S. grandifolia* with red color similar with tanshinones. | S3 |
| **Fig. S2-S3** | The total ion chromatography (TIC) of *S. miltiorrhiza* and *S. grandifolia* root tissue | S3-S4 |
| **Fig. S4-S9** | The MS/MS spectrum and the fragmentation pathway of salvianolic acid B, rosmarinic acid, lithospermic acid, cryptotanshinone, tanshinone IIA, dihydrotanshinone I. | S4-S7 |
| **Fig. S10** | Heat map of metabolomics and chemometrics clustering of *S. miltiorrhiza* and *S. grandifolia*. | S8 |
| **Fig. S11** | Principal component analysis of *S. miltiorrhiza* and *S. grandifolia* roots. | S9 |
| **Fig. S12** | The surface of the slide with red and black marker marks evenly distributed as the sample | S9 |
| **Fig. S13** | Best signal under positive and negative ions. | S9 |
| **Fig. S14** | Positive and negative DESI-MS spectra of 3mm spots of standard on the surface of glass slide. | S10 |
| **Fig. S15** | Imaging of *S. miltiorrhiza* standard under negative ions. | S10 |
| **Fig. S16** | Functional annotation of the transcriptome (KEGG). | S11 |
| **Table S1** | Representative constituents of *S. miltiorrhiza* and *S. grandifolia* phytochemical research private database. | S11-S13 |
| **Table S2** | Characterization of major metabolites in *Salvia miltiorrhiza Bge.* (SM) and *Salvia grandifolia W. W. Smith* (SG) root tissues by LC-MS/MS. | S14-17 |
| **Table S3** | Selected metabolites putatively assigned in *S. miltiorrhiza* (SM) and *S. grandifolia* (SG) root tissues by DESI MSI. | S18-S20 |
| **Table S4** | Differential expressed transcriptome gene in SG and SM. | S22 |
| **Table S5** | Differential genome functional annotation (KEGG) top 20. | S22 |
| **Table S6** | List of oligonucleotide primer sequences. | S22-23 |

**Fig. S1.** The crude drug of *S. miltiorrhiza* and *S. grandifolia* with red color similar with tanshinones. Cultivation photos of *Salvia miltiorrhiza* (A a) and *Salvia grandifolia* W. W. Smith (A b), Wild *Salvia grandifolia* W. W. Smith Photos(A c); Reference standards miltirone (B a) and dihydrotanshinone I (B b); 70% methanol extract of *Salvia miltiorrhiza* (C).

**Fig. S2.** The total ion chromatography (TIC) of *S. miltiorrhiza* root tissue.

**Fig. S3.** The total ion chromatography (TIC) of *S. grandifolia* root tissue

**Fig. S4.**The MS/MS spectrum and the fragmentation pathway of salvianolic acid B from *S. miltiorrhiza.*

**Fig. S5.** The MS/MS spectrum and the fragmentation pathway of dihydrotanshinone I from *S. miltiorrhiza.*

 **Fig. S6**. The MS/MS spectrum and the fragmentation pathway of rosmarimic acid from *S. miltiorrhiza.*

**Fig. S7**. The MS/MS spectrum and the fragmentation pathway of lithospermic acid from *S. miltiorrhiza.*

 **Fig. S8**. The MS/MS spectrum and the fragmentation pathway of cryptotanshinone from *S. miltiorrhiza.*

**Fig. S9**. The MS/MS spectrum and the fragmentation pathway of tanshinone ⅡA from *S. miltiorrhiza.*

**Fig. S10**. Heat map of metabolomics and chemometrics clustering of *S. miltiorrhiza* and *S. grandifolia*.

**Fig. S11**. Principal component analysis of *S. miltiorrhiza* and *S. grandifolia* roots.

**Fig. S12**. The surface of the slide with red and black marker marks evenly distributed as the sample.

Optimal signal strength in positive ion mode Optimal signal strength in negative ion mode

**Fig. S13**. Best signal under positive and negative ions.

**Fig. S14.** Positive and negative DESI-MS spectra of 3mm spots of standard on the surface of glass slide.

**Fig. S15.** Imaging of *S. miltiorrhiza* standard under negative ions. (A. B, C and D represent 70% methanol, 80% methanol, 80% methanol + 0.1% formic acid, 90% methanol + 0.1% formic acid respectively; 1, 2 and 3 represent three sample repetitions.)

**Fig. S16.** Functional annotation of the transcriptome (KEGG).

**Table S1.** Representative constituents of *S. miltiorrhiza* and *S. grandifolia* phytochemical research private database.

| No. | Compound Name | Molecular Fomular | Theoretical m/z | No. | Compound Name | Molecular Fomular | Theoretical m/z |
| --- | --- | --- | --- | --- | --- | --- | --- |
| 1 | Protocatechualdehyde | C7H6O3 | 138.0317 | 66 | Danshenol A | C21H20O4 | 336.1362 |
| 2 | Phthalic Acid | C8H6O4 | 166.0266 | 67 | methyltanshinonate | C20H18O5 | 338.1154 |
| 3 | Caffeic acid | C9H8O4 | 180.0423 | 68 | salvianolic acid G | C18H12O7 | 340.0583 |
| 4 | Ethyl 4-Hydroxyphenylacetate | C10H12O3 | 180.0786 | 69 | salvianolic acid G I isomer | C18H12O7 | 340.0583 |
| 5 | Ferulic acid | C10H10O4 | 194.0579 | 70 | salvianolic acid G II isomer | C18H12O7 | 340.0583 |
| 6 | Methyl Caffeate | C10H10O4 | 194.0579 | 71 | trijuganone C | C20H20O5 | 340.1311 |
| 7 | Danshensu/Salvianic acid A | C9H10O5 | 198.0528 | 72 | Anacardic acid C15:3 | C22H30O3 | 342.2195 |
| 8 | chrysene | C18H12 | 228.0939 | 73 | Royleanone-4 | C18H21O4 | 344.1990 |
| 9 | Tanshinlactone | C17H12O3 | 264.0786 | 74 | Royleanonic acid | C20H26O5 | 346.1780 |
| 10 | 3,4-Phenanthrenedione | C18H16O2 | 264.1229 | 75 | Anacardic acid | C22H36O3 | 348.2664 |
| 11 | 4-methylenemiltirone | C18H18O2 | 266.1124 | 76 | przewalskinic acid | C18H14O8 | 358.0689 |
| 12 | norsalvioxide | C18H24O2 | 272.1776 | 77 | Rosmarinic acid | C18H16O8 | 360.0845 |
| 13 | Isotanshinone I | C18H12O3 | 276.0786 | 78 | Micranthin B | C22H28O5 | 372.1937 |
| 14 | Tanshinone I | C18H12O3 | 276.0786 | 79 | 7α-Acetoxyroyleanone | C22H30O5 | 374.2093 |
| 15 | 1,2-Dihydrotanshinquinone | C18H14O3 | 278.0943 | 80 | Ethyl rosmarinate | C20H20O8 | 388.1158 |
| 16 | Dihydrotanshinone I | C18H14O3 | 278.0943 | 81 | DEHP | C24H38O4 | 390.2770 |
| 17 | Methylenetanshinquinone | C18H14O3 | 278.0943 | 82 | Salvianolic acid D | C20H18O10 | 418.0900 |
| 18 | Dibutyl phthalate | C16H22O4 | 278.1518 | 83 | Olenolic acid | C30H48O3 | 456.3603 |
| 19 | 1,2- dihydrotanshinone I | C18H15O3 | 280.1099 | 84 | Hyptadienic Acid | C30H46O4 | 470.3396 |
| 20 | 15, 16-dihydrotanshinone I | C18H15O3 | 280.1099 | 85 | Corosolic Acid | C30H48O4 | 472.3553 |
| 21 | Danshenxinkun B/Neotanshinone B | C18H16O3 | 280.1099 | 86 | Tormentic acid | C30H48O5 | 488.3502 |
| 22 | Methylenedihydrotanshinquinone | C18H16O3 | 280.1099 | 87 | Isosalvianolic acid C | C26H20O10 | 492.1056 |
| 23 | Tetrahydro tanshinone I | C18H16O3 | 280.1099 | 88 | Salvianolic acid C | C26H20O10 | 492.1056 |
| 24 | Trijuganone B | C18H16O3 | 280.1099 | 89 | Salvianolic acid A | C26H22O10 | 494.1213 |
| 25 | 16-Hydroxy-Ro 09-0680 | C18H16O3 | 280.1178 | 90 | feruloyl-di-glucoside | C22H30O14 | 518.1636 |
| 26 | 1,2-Didehydromiltirone | C19H20O2 | 280.1463 | 91 | rosmarinic acid glucoside | [C24H26O13](https://pubchem.ncbi.nlm.nih.gov/) | 522.1373 |
| 27 | Dehydromiltirone | C19H20O2 | 280.1463 | 92 | grandifolione | C30H40O8 | 528.2723 |
| 28 | Miltirone | C19H22O2 | 282.1620 | 93 | 1-Hydroxy pinoresinol-1-O β- D -glucoside | C26H32O12 | 536.1894 |
| 29 | ferruginol | C20H30O | 286.2297 | 94 | Lithospermic acid | C27H22O12 | 538.1111 |
| 30 | 1,2-Didehydrotanshinone IIA | C19H16O3 | 292.1099 | 95 | salvianolic acid H | C27H22O12 | 538.1111 |
| 31 | Trijuganone A | C18H14O4 | 294.0892 | 96 | Salvianolic acid I | C27H22O12 | 538.1111 |
| 32 | Isotanshinone ⅡA | C19H18O3 | 294.1256 | 97 | Salvianolic acid K | C27H24O13 | 556.1217 |
| 33 | Tanshinone IIA | C19H18O3 | 294.1256 | 98 | Daucosterol | C35H60O6 | 576.4390 |
| 34 | przewaquinone C | C18H16O4 | 296.1049 | 99 | salvianolic acid G-danshensu-glucoside | C33H30O16 | 682.1534 |
| 35 | Tanshindiol B | C18H16O5 | 312.3170 | 100 | salvianolic acid G-danshensu-glucoside | C33H30O16 | 682.1534 |
| 36 | Tanshinone-VI | C18H16O4 | 296.1049 | 101 | rosmarinic acid di-glucoside | C30H36O18 | 684.1902 |
| 37 | DanshenXinkun A | C18H16O4 | 296.1132 | 102 | didehydrosalvianolic acid B | C36H28O16 | 716.1377 |
| 38 | Cryptotanshinone | C19H20O3 | 296.1412 | 103 | przewalskinic acid di-danshensu | C36H30O16 | 718.1534 |
| 39 | Isocryptotanshinone | C19H20O3 | 296.1412 | 104 | Salvianolic acid B、Isosalvianolic acid B | C36H30O16 | 718.1534 |
| 40 | Deoxyneocryptotanshinone | C19H22O3 | 298.1646 | 105 | salvianolic acid E | C36H30O16 | 718.1534 |
| 41 | miltipolone | C19H24O3 | 300.1725 | 106 | Salvianolic acid L | C36H30O16 | 718.1534 |
| 42 | Sugiol | C20H28O2 | 300.2089 | 107 | 3′-O-Monomethyllith- ospermic acid B | C37H32O16 | 732.1690 |
| 43 | grandifoliol | C19H26O3 | 302.1882 | 108 | 9-methyl salvianolate B | C37H32O16 | 732.1690 |
| 44 | 11-Hydroxy-ferruginol | C20H30O2 | 302.2168 | 109 | methoxyl-salvianolic acid B | C37H32O16 | 732.1690 |
| 45 | Tanshinaldehyde II | C19H18O4 | 308.1049 | 110 | Monomethyl Lithospermate B | C36H28MgO16 | 740.1228 |
| 46 | Przewatanshinone A | C18H14O5 | 310.0841 | 111 | Dimethyllithospermate B | C38H34O16 | 746.1847 |
| 47 | Hydroxytanshinone | C19H18O4 | 310.1205 | 112 | Verbascose | C30H52O26 | 828.2747 |
| 48 | Tanshinaldehyde | C19H18O4 | 310.1205 | 113 | salvianolic acid B glucoside | C42H40O21 | 880.2062 |
| 49 | Tanshinone IIA anhydride | C19H18O4 | 310.1205 | 114 | nepetoidin B | C17H14O6 | 314.0790 |
| 50 | Tanshinone IIB | C19H18O4 | 310.1205 | 115 | grandifolia A | C19H26O5 | 334.1780 |
| 51 | Tanshindiol C | C18H16O5 | 312.0998 | 116 | grandifolia B | C19H24O3 | 300.1725 |
| 52 | przewaquinonate A | C19H20O4 | 312.1362 | 117 | grandifolia C | C18H26O2 | 274.1933 |
| 53 | Salvianolic acid F | C17H14O6 | 314.0790 | 118 | grandifolia D | C19H22O3 | 298.1569 |
| 54 | hydroxytanshinone VI | C18H18O5 | 314.1154 | 119 | grandifolia E | C21H18O6 | 366.1103 |
| 55 | neocryptotanshinone | [C19H22O4](http://www.chemicalbook.com/javascript:showMsgDetail('ProductSynonyms.aspx?CBNumber=CB23037421&postData3=CN&SYMBOL_Type=A');) | 314.1518 | 120 | grandifolia F | C22H24O6 | 384.1573 |
| 56 | 6,7-Dehydroroyleanone | C20H26O3 | 314.1882 | 121 | grandifolia H | C36H30O12 | 654.1737 |
| 57 | 11-Hydroxy-sugiol | C20H28O3 | 316.1960 | 122 | grandifolia I | C32H32O16 | 672.1690 |
| 58 | 10-hydroxymethyltetrahydromiltirone | C20H28O3 | 316.1960 | 123 | grandifolia G | C19H20O5 | 328.1311 |
| 59 | 20-Hydroxy-sugiol | C20H28O3 | 316.1960 | 124 | grandifoliol | C19H26O3 | 302.18819 |
| 60 | 11，20-dihydroxy-ferruginol | C20H30O3 | 318.2117 | 125 | grandifolione | C19H24O3 | 300.17254 |
| 61 | 7-hydroxyallyl-royleanone | C20H26O4 | 330.1831 | 126 | β-daucosterol | C35H60O6 | 576.43899 |
| 62 | Carnosol | C20H26O4 | 330.2000 | 127 | β-Sitosterol | C29H50O | 414.38617 |
| 63 | 11，20-dihydroxy-sugiol | C20H28O4 | 332.1909 | 128 | norsalvioxide | C18H24O2 | 272.17763 |
| 64 | Horminone | C20H28O4 | 332.1988 | 129 | 2α-Hydroxyursolic acid | C30H48O4 | 472.35526 |
| 65 | Carnosic acid | C20H28O4 | 332.2021 | 130 | chrysene | C18H12O3 | 276.07864 |

**Table S2 Characterization of major metabolites in *Salvia miltiorrhiza* Bge*.* (SM) and *Salvia grandifolia* W. W. Smith (SG) root tissues by LC-MS/MS.**

| No. | Compound Name | Molecular Fomular | Adduct Ion | Theoretical m/z | RT | Measured m/z | | No. | Compound Name | Molecular Fomular | Adduct Ion | Theoretical m/z | RT | Measured m/z | |
| --- | --- | --- | --- | --- | --- | --- | --- | --- | --- | --- | --- | --- | --- | --- | --- |
|  |  |  |  |  |  | SM | SG |  |  |  |  |  |  | SM | SG |
| 1 | Tanshinone ⅡA | C19H18O3 | [M+H]+ | 295.1334 | 50.53 | 295.1329 | 295.0922 | 39 | Tanshinone IIB | C19H18O4 | [M+H]+ | 311.1283 | 37.88 | 311.1283 | 311.1283 |
| 2 | cryptotanshinone | C19H20O3 | [M+H]+ | 297.1491 | 48.18 | 297.1493 | 297.1438 | 40 | Tanshindiol C | C18H16O5 | [M+H]+ | 313.1076 | 24.44 | 313.1076 | 313.1076 |
| 3 | 1,2-Dehydromiltirone | C19H20O2 | [M+H]+ | 281.1541 | 43.69 | 281.1779 | 281.1779 | 41 | Danshenol A/C | C21H20O4 | [M+H]+ | 337.14398 | 44.32 | 337.1440 | 337.1440 |
| 4 | tetrahydrotanshinone | C18H16O3 | [M+Na]+ | 303.3097 | 46.06 | 303.0971 | 303.3097 | 42 | Methyl tanshinonate | C20H18O5 | [M+H]+ | 339.12325 | 46.96 | 339.1233 | 339.1233 |
| 5 | dihydroisotanshinone 1 | C18H14O3 | [M+Na]+ | 301.2897 | 45.01 | 301.0779 | 301.0758 | 43 | trijuganone C | C20H20O5 | [M+H]+ | 341.1389 | 40.22 | 341.1389 | 341.1389 |
| 6 | miltirone | C19H22O2 | [M+Na]+ | 305.1517 | 50.97 | 305.1549 | 305.1541 | 44 | Ferulic acid | C10H10O4 | [M-H]- | 193.05008 | 18.02 | 193.0501 | 193.0501 |
| 7 | przewaquinone B | C18H12O4 | [M+Na]+ | 315.0633 | 46.54 | 315.1179 | 315.1489 | 45 | Salvianolic acid F | C17H14O6 | [M-H]- | 313.0707 | 13.43 | 313.0707 | 313.0707 |
| 8 | tanshinlactone | C17H12O3 | [M]+ | 264.0786 | 50.35 | 264.0773 | 264.0631 | 46 | salvianolic acid G | C18H12O7 | [M-H]- | 339.0501 | 18.48 | 339.0501 | 339.0501 |
| 9 | methyl rosmarina | C19H18O8 | [M+Na]+ | 397.09 | 47.06 | 397.2832 | 397.1948 | 47 | przewalskinic acid | C18H14O8 | [M-H]- | 357.06104 | 12.23 | 357.0610 | 357.0610 |
| 10 | protocatechualdehyde | C7H6O3 | [M-H]- | 137.0239 | 5.13 | 137.0239 | 137.0356 | 48 | Rosmarinic acid | C18H16O8 | [M-H]- | 359.07669 | 20.5 | 359.0767 | 359.0767 |
| 11 | caffeic acid | C9H8O4 | [M-H]- | 179.0344 | 8.02 | 179.0441 | 179.0598 | 49 | Salvianolic acid D | C20H18O10 | [M-H]- | 417.08217 | 19.01 | 417.0822 | 417.0822 |
| 12 | rosmarinic acid glucoside | C24H26O13 | [M-H]- | 521.1295 | 17.78 | 521.1295 | 521.1774 | 50 | Lithospermic acid | C27H22O12 | [M-H]- | 537.1033 | 21.63 | 537.1033 | 537.1033 |
| 13 | epirosmanol | C20H26O5 | [M-H]- | 345.1702 | 33.75 | 345.1702 | 345.1792 | 51 | Salvianolic acid H/I | C27H22O12 | [M-H]- | 537.1033 | 18.4 | 537.1033 | 537.1033 |
| 14 | isorosmanol | C20H26O5 | [M-H]- | 345.1702 | 49.23 | 345.1702 | 345.1792 | 52 | Salvianolic acid J | C27H22O12 | [M-H]- | 537.1033 | 14.7 | 537.1033 | 537.1033 |
| 15 | 7α-Acetoxyroyleanone | C22H30O5 | [M-H]- | 373.2112 | 51.36 | 373.2112 | 373.2134 | 53 | Methyl salvianolate H/I | C28H24O12 | [M-H]- | 551.11895 | 30.5 | 551.1190 | 551.1190 |
| 16 | 11-Hydroxy-sugiol | C20H28O3 | [M-H]- | 315.1973 | 44.98 | 315.1922 | 315.2038 | 54 | didehydrosalvianolic acid B | C36H28O16 | [M-H]- | 715.12991 | 24.95 | 715.1299 | 715.1299 |
| 17 | salvianolic acid A | C26H22O10 | [M-H]- | 493.1135 | 21.98 | 492.1848 | 493.1135 | 55 | Salvianolic acid B | C36H30O16 | [M-H]- | 717.14556 | 23.45 | 717.1456 | 717.1456 |
| 18 | danshensu | C9H10O5 | [M-H]- | 197.0682 | 2.22 | 197.0682 | 197.0682 | 56 | Methoxyl-salvianolic acid B | C37H32O16 | [M-H]- | 731.16121 | 26.05 | 731.1612 | 731.1612 |
| 19 | protocatechuic acid A | (HO)2C6H3COOH | [M-H]- | 153.0188 | 26.08 | 153.9738 | 153.0188 | 57 | salvianolic acid B glucoside | C42H40O21 | [M-H]- | 879.19838 | 20.79 | 879.1984 | 879.1984 |
| 20 | cinnamic acid | C9H8O2 | [M-H]- | 147.0446 | 9.91 | 146.9658 | 146.9660 | 58 | 10-hydroxymethyl tetrahydromiltirone | C20H28O3 | [M-H]- | 315.19602 | 48.31 | 315.1960 | 315.1960 |
| 21 | ursolic acid | C30H48O3 | [M-H]- | 455.3525 | 47.37 | 455.3004 | 455.3525 | 59 | Tanshinone I isomer1 | C18H12O3 | [M+H]+ | 277.08647 | 38.70 | 277.0865 | 277.0865 |
| 22 | przewaquinonate A | C19H20O4 | [M-H]- | 311.1283 | 45.68 | 311.2226 | 311.1283 | 60 | Tanshinone I isomer2 | C18H12O3 | [M+H]+ | 277.08647 | 49.72 | 277.0865 | 277.0865 |
| 23 | hydroxytanshinone VI | C18H18O5 | [M-H]- | 313.107 | 46.53 | 313.1447 | 313.1070 | 61 | Dihydrotanshinone I isomer1 | C18H14O3 | [M+H]+ | 279.10212 | 44.96 | 279.1021 | 279.1021 |
| 24 | trijuganone A | C18H14O4 | [M-H]- | 293.0819 | 36.52 | 293.2119 | 293.0819 | 62 | Dihydrotanshinone I isomer2 | C18H14O3 | [M+H]+ | 279.10212 | 49.59 | 279.1021 | 279.1021 |
| 25 | 1,2- dihydrotanshinone I/15, 16-dihydrotanshinone I | C18H15O3 | [M-H]- | 279.102 | 50.83 | 279.2329 | 279.1020 | 63 | Trijuganone B | C18H16O3 | [M+H]+ | 281.11777 | 45.59 | 281.1178 | 281.1178 |
| 26 | grandifolia C | C18H26O2 | [M+Na]+ | 297.183 | 50.45 | 297.1830 | 297.1828 | 64 | DanshenXinkun A isomer | C18H16O4 | [M+H]+ | 297.11268 | 41.58 | 297.1127 | 297.1127 |
| 27 | grandifolia D | C19H22O3 | [M+H]+ | 299.1647 | 53.44 | 299.1647 | 299.1955 | 65 | Tanshinaldehyde isomer1 | C19H16O4 | [M+H]+ | 309.11268 | 42.33 | 309.1127 | 309.1127 |
| 28 | grandifolione/grandifolia B | C19H24O3 | [M+H]+ | 301.1803 | 46.55 | 301.1803 | 301.2169 | 66 | Tanshindiol C isomer1 | C18H16O5 | [M+H]+ | 313.10760 | 27.12 | 313.1076 | 313.1076 |
| 29 | grandifolia G | C19H20O5 | [M+H]+ | 329.1389 | 47.36 | 329.1389 | 329.1821 | 67 | Tanshindiol C isomer2 | C18H16O5 | [M+H]+ | 313.10760 | 33.40 | 313.1076 | 313.1076 |
| 30 | nepetoidin B | C17H14O6 | [M-H]- | 313.0712 | 46.44 | 313.0712 | 313.1892 | 68 | trijuganone C isomer | C20H20O5 | [M+H]+ | 341.13890 | 51.80 | 341.1389 | 341.1389 |
| 31 | grandifolia I | C32H32O16 | [M-H]- | 671.1612 | 55.47 | 671.1612 | 671.4831 | 69 | 11,20-dihydroxy sugiol | C20H28O4 | [M+H]+ | 331.1909 | 49.8 | 331.1909 | 331.1909 |
| 32 | salvinone | C18H20O2 | [M+H]+ | 269.1541 | 49.19 | 269.1541 | 269.1938 | 70 | 11-hydroxy ferruginol | C20H30O2 | [M+H]+ | 301.2168 | 52.68 | 301.2168 | 301.2168 |
| 33 | grandifoliol | C19H26O3 | [M+H]+ | 303.1960 | 44.86 | 303.1479 | 303.1480 | 71 | 11,20-dihydroxy ferruginol | C20H30O3 | [M+H]+ | 317.2118 | 50.42 | 317.2118 | 317.2118 |
| 34 | 4-Methylenemiltirone | C17H12O3 | [M+H]+ | 265.08647 | 49.9 | 265.0865 | 265.0865 | 72 | Salvianolic acid F isomer1 | C17H14O6 | [M-H]- | 313.0707 | 28.57 | 313.0707 | 313.0707 |
| 35 | 1,2-Didehydrotanshinone IIA | C19H16O3 | [M+H]+ | 293.1178 | 49.64 | 293.1178 | 293.1178 | 73 | Salvianolic acid F isomer2 | C17H14O6 | [M-H]- | 313.07121 | 30.82 | 313.0712 | 313.0712 |
| 36 | DanshenXinkun A | C18H16O4 | [M+H]+ | 297.11268 | 33.76 | 297.1127 | 297.1127 | 74 | Salvianolic acid G isomer | C18H12O7 | [M-H]- | 339.05048 | 29.55 | 339.0505 | 339.0505 |
| 37 | Isocryptotanshinone | C19H20O3 | [M+H]+ | 297.1491 | 45.51 | 297.1491 | 297.1491 | 75 | 8-Hydroxy-9''-methyl-, salvianolate B | C37H34O17 | [M-H]- | 749.17177 | 23.52 | 749.1718 | 749.1718 |
| 38 | Sugiol | C20H28O2 | [M+H]+ | 301.2168 | 48.77 | 301.2168 | 301.2168 | 76 | salvianolic acid B glucoside isomer1 | C42H40O21 | [M-H]- | 879.19838 | 18.67 | 879.1984 | 879.1984 |

**Table S3 Selected metabolites putatively assigned in *Salvia miltiorrhiza* Bge. (SM) and *Salvia grandifolia* W. W. Smith (SG) root tissues by DESI MSI**

| No. | Compound Name | Molecular Fomular | Adduct Ion | Theoretical m/z | Measured m/z | | HDI max intensity | | Salvia miltiorrhiza Bge. (SM) | | | Salvia grandifolia W. W. Smith (SG) | | |
| --- | --- | --- | --- | --- | --- | --- | --- | --- | --- | --- | --- | --- | --- | --- |
|  |  |  |  |  | SM | SG | SM | SG | cortex | phloem | xylem | cortex | phloem | xylem |
| 1 | tanshinoneⅡA | C19H18O3 | [M+H]+ | 295.1334 | 295.1329 | 295.0922 | 0.2987 | 0.01765 | ＋ | ＋ | － | ＋ | － | － |
| 2 | cryptotanshinone | C19H20O3 | [M+H]+ | 297.1491 | 297.1493 | 297.1438 | 0.10346 | 0.02001 | ＋ | ＋ | － | ＋ | ＋ | － |
| 3 | 1,2-Dehydromiltirone | C19H20O2 | [M+H]+ | 281.1541 | 281.1779 | 281.1779 | 0.0296 | 0.01279 | ＋ | ＋ | － | ＋ | － | － |
| 4 | tetrahydrotanshinone | C18H16O3 | [M+H]+ | 303.3097 | 303.0971 | － | 0.06328 | － | ＋ | ＋ | － | － | － | － |
| 5 | dihydroisotanshinone | C18H14O3 | [M+Na]+ | 301.2897 | 301.0779 | 301.0758 | 0.04416 | 0.01993 | ＋ | ＋ | － | ＋ | ＋ | － |
| 6 | miltirone | C19H22O2 | [M+Na]+ | 305.1517 | 305.1549 | 305.1541 | 0.02826 | 0.04346 | ＋ | ＋ | － | ＋ | ＋ | － |
| 7 | 10-hydroxymerhyl tetrahydromiltirone | C20H28O3 | [M+H]+ | 317.2116 | 317.0559 | 317.2101 | 0.0633 | 0.02195 | ＋ | ＋ | － | ＋ | ＋ | － |
| 8 | przewaquinone B | C18H12O4 | [M+Na]+ | 315.0633 | 315.1179 | 315.1489 | 0.03693 | 0.01773 | － | － | ＋ | ＋ | ＋ | ＋ |
| 9 | 17-hydroxytanshindiol | C18H18O5 | [M+Na]+ | 337.1052 | 337.1022 | 337.1588 | 0.03258 | 0.01429 | ＋ | ＋ | ＋ | ＋ | － | ＋ |
| 10 | danshenxinkun D | C21H20O4 | [M]+ | 336.1362 | 336.1129 | 336.1294 | 0.05382 | 0.01167 | ＋ | － | － | ＋ | － | － |
| 11 | tanshinlactone | C17H12O3 | [M]+ | 264.0786 | 264.0773 | 264.0631 | 0.0823 | 0.01310 | － | ＋ | － | ＋ | ＋ | ＋ |
| 12 | methyl rosmarina | C19H18O8 | [M+Na]+ | 397.0900 | 397.2832 | 397.1948 | 0.02361 | 0.06678 | － | ＋ | － | ＋ | ＋ | ＋ |
| 13 | protocatechualdehyde | C7H6O3 | [M-H]- | 137.0239 | 137.0317 | 137.0356 | 0.00754 | 0.01477 | ＋ | ＋ | ＋ | ＋ | ＋ | ＋ |
| 14 | caffeic acid | C9H8O4 | [M-H]- | 179.0344 | 179.0441 | 179.0598 | 0.01208 | 0.01645 | ＋ | ＋ | ＋ | ＋ | ＋ | ＋ |
| 15 | rosmarinic acid glucoside | C24H26O13 | [M-H]- | 521.1295 | － | 521.1774 | － | 0.02499 | － | － | － | － | ＋ | － |
| 16 | epirosmanol/isorosmanol | C20H26O5 | [M-H]- | 345.1702 | － | 345.1792 | － | 0.02104 | － | － | － | ＋ | － | － |
| 17 | 7α-Acetoxyroyleanone | C22H30O5 | [M-H]- | 373.2112 | － | 373.2134 | － | 0.030369 | － | － | － | － | ＋ | － |
| 18 | 11-Hydroxy-sugiol | C20H28O3 | [M-H]- | 315.1973 | 315.1922 | 315.2038 | 0.05288 | 0.06939 | ＋ | ＋ | － | ＋ | ＋ | － |
| 19 | 11,20-dihydroxy-sugiol | C20H28O4 | [M-H]- | 331.1914 | 331.1913 | 331.1913 | 0.02039 | 0.02039 | ＋ | ＋ | － | ＋ | ＋ | － |
| 20 | 11,20-dihydroxy-ferruginoll | C20H30O3 | [M-H]- | 317.2126 | 317.2115 | 317.2162 | 0.03895 | 0.0154 | ＋ | － | － | ＋ | ＋ | － |
| 21 | salvianolic acid C | C26H20O10 | [M-H]- | 491.0978 | 491.3419 | － | 0.01569 | － | ＋ | ＋ | ＋ | － | － | － |
| 22 | salvianolic acid A | C26H22O10 | [M-H]- | 493.1135 | 493.2727 | － | 0.00567 | － | ＋ | ＋ | ＋ | － | － | － |
| 23 | danshensu | C9H10O5 | [M-H]- | 197.0682 | 197.9631 | － | 0.01996 | － | ＋ | ＋ | ＋ | － | － | － |
| 24 | protocatechuic acid A | (HO)2C6H3COOH | [M-H]- | 153.0188 | 153.9738 | － | 0.00667 | － | ＋ | ＋ | ＋ | － | － | － |
| 25 | przewalskinic acid | C18H14O8 | [M-H]- | 357.0615 | 357.1956 | － | 0.0074 | － | ＋ | ＋ | ＋ | － | － | － |
| 26 | cinnamic acid | C9H8O2 | [M-H]- | 147.0446 | 146.9658 | 146.966 | 0.22428 | 0.05981 | ＋ | － | ＋ | ＋ | － | ＋ |
| 27 | ursolic acid | C30H48O3 | [M-H]- | 455.3525 | 455.3004 | － | 0.00516 | － | ＋ | ＋ | ＋ | － | － | － |
| 28 | przewaquinonate A | C19H20O4 | [M-H]- | 311.1283 | 311.2226 | － | 0.08507 | － | ＋ | ＋ | － | － | － | － |
| 29 | hydroxytanshinone VI | C18H18O5 | [M-H]- | 313.1070 | 313.1447 | － | 0.02933 | － | ＋ | － | － | － | － | － |
| 30 | trijuganone A | C18H14O4 | [M-H]- | 293.0819 | 293.2119 | － | 0.04676 | － | ＋ | ＋ | － | － | － | － |
| 31 | 1,2- dihydrotanshinone I/15, 16-dihydrotanshinone I | C18H15O3 | [M-H]- | 279.102 | 279.2329 | － | 0.15014 | － | ＋ | ＋ | － | － | － | － |
| 32 | grandifolia C | C18H26O2 | [M+Na]+ | 297.183 | － | 297.1828 | － | 0.0155 | － | － | － | ＋ | ＋ | － |
| 33 | grandifolia D | C19H22O3 | [M+H]+ | 299.1647 | － | 299.1955 | － | 0.03566 | － | － | － | ＋ | ＋ | － |
| 34 | grandifolione/grandifolia B | C19H24O3 | [M+H]+ | 301.1803 | － | 301.2169 | － | 0.03688 | － | － | － | ＋ | ＋ | － |
| 35 | grandifolia G | C19H20O5 | [M+H]+ | 329.1389 | － | 329.1821 | － | 0.02348 | － | － | － | ＋ | ＋ | － |
| 36 | nepetoidin B | C17H14O6 | [M-H]- | 313.0712 | － | 313.1892 | － | 0.03671 | － | － | － | ＋ | ＋ | － |
| 37 | grandifolia I | C32H32O16 | [M-H]- | 671.1612 | － | 671.4831 | － | 0.05205 | － | － | － | ＋ | ＋ | － |
| 38 | salvinone | C18H20O2 | [M+H]+ | 269.1541 | － | 269.1938 | － | 0.02324 | － | － | － | ＋ | ＋ | － |
| 39 | grandifoliol | C19H26O3 | [M+H]+ | 303.1960 | 303.1479 | 303.148 | 0.00967 | 0.01667 | － | ＋ | ＋ | － | ＋ | ＋ |

**Notes : +:Detected -:Not Detected**

**Table S4. Differential expressed transcriptome gene in SG and SM.**

|  | Total difference | High-expression in Sg | High-expression in Sm |
| --- | --- | --- | --- |
| Transcripts | 132,776 | 69,969 | 62,807 |
| CYP | 271 | 164 | 107 |

**Table S5.** Differential genome functional annotation (KEGG) top 20.

| pathway_term | rich_factor | q_value | gene_number |
| --- | --- | --- | --- |
| Plant hormone signal transduction | 0.207437348 | 5.58E-87 | 1283 |
| Amino sugar and nucleotide sugar metabolism | 0.155776173 | 1.67E-20 | 863 |
| Starch and sucrose metabolism | 0.15261959 | 3.18E-20 | 938 |
| Basal transcription factors | 0.185787274 | 1.12E-19 | 400 |
| SNARE interactions in vesicular transport | 0.189558732 | 3.83E-16 | 305 |
| Terpenoid backbone biosynthesis | 0.168174962 | 2.02E-15 | 446 |
| Glycosylphosphatidylinositol(GPI)-anchor biosynthesis | 0.203787196 | 5.33E-15 | 226 |
| Circadian rhythm - plant | 0.188208617 | 3.85E-13 | 249 |
| Sesquiterpenoid and triterpenoid biosynthesis | 0.213592233 | 8.67E-12 | 154 |
| Phenylpropanoid biosynthesis | 0.146716233 | 1.07E-10 | 592 |
| Glycerophospholipid metabolism | 0.145687364 | 1.73E-10 | 603 |
| Inositol phosphate metabolism | 0.148353339 | 1.89E-09 | 482 |
| Nucleotide excision repair | 0.152207591 | 5.70E-09 | 393 |
| Carotenoid biosynthesis | 0.182015168 | 2.41E-08 | 168 |
| Porphyrin and chlorophyll metabolism | 0.157239819 | 1.15E-07 | 278 |
| Galactose metabolism | 0.143864146 | 1.55E-07 | 449 |
| Glycosaminoglycan degradation | 0.187683284 | 3.17E-07 | 128 |
| Ubiquitin mediated proteolysis | 0.129745119 | 4.93E-07 | 957 |
| Cell cycle - Caulobacter | 0.185507246 | 4.99E-07 | 128 |
| Photosynthesis | 0.173719376 | 7.85E-07 | 156 |

**Table S6.** List of oligonucleotide primer sequences.

| qRTSmActin-F | GGTGCCCTGAGGTCCTGTT |
| --- | --- |
| qRTSmActin-R | AGGAACCACCGATCCAGACA |
| qRTSmCPS1-F | TTCCTCGAGGGATTCGACAG |
| qRTSmCPS1-R | TTCTTCCCTGAAGGCGATGT |
| qRTSmCYP76AK1-F | AAGCGCGGGTGAATGAATAC |
| qRTSmCYP76AK1-R | ACGGAATCAGCTCGAAATGC |
| qRTSmCYP76AK5-F | GGAGAAGCTGAAAGCAGAGC |
| qRTSmCYP76AK5-R | TGCTGCATGAGGAGCTAAGA |
| qRTSmDXS1-F | ACATCGGTCCAGTTGATGGT |
| qRTSmDXS1-R | GCTGGATCGAACTTGGTCAC |
| qRTSmDXS2-F | GCCGCCTAACAACAAAGGAA |
| qRTSmDXS2-R | TTGCAGAATCTCGCATCAGC |
| qRTSmKSL1-F | CTTTCGCCGTGAAATGCAAC |
| qRTSmKSL1-R | AGTATCGGTCGACTCCCAAC |
| qRTSmDXR-F | CTGGAGGACCTTTCGTCCTT |
| qRTSmDXR-R | CAACCGGCAAATCCCTGAAA |
| qRTSmTⅡAS-F | ACCGATCCTTGTTTCCTAAC |
| qRTSmTⅡAS-R | CCGATTTGCGTGGACTCTAT |
| qRTSmCYP71D375-F | CGGAGACGCTCTCCTACAAT |
| qRTSmCYP7D375-R | ATGCATGCTCTCCTCCTCTC |
| qRTSmCYP76AH1-F | ATGCGCCAAAGAAGGATGAC |
| qRTSmCYP76AH1-R | GGGTTCATCACTAGCTCCGA |
| qRTSmCYP76AH3-F | AAGGGACTCAGGTGCTCATC |
| qRTSmCYP76AH3-R | GGAAGGCGTCTGGATTGTTC |
| qRTSgActin-F | CAGAGCGTGGCTACATGTTC |
